# Strategic Design of Aptamer‐Guided Aggregation‐Induced Emission Nanoparticles for Targeted Photodynamic Therapy in Breast Cancer

**DOI:** 10.1002/advs.202503358

**Published:** 2025-09-09

**Authors:** Charlie C. H. Ma, Qingqing Liu, Yue Sui, Wutong Du, Kristy W. K. Lam, Jacky W. Y. Lam, Chao Li, Tengteng Chen, Jianwei Sun, Nuomin Li, Ryan T. K. Kwok, Jianping Chen, Feiyi Sun, Ben Zhong Tang

**Affiliations:** ^1^ Department of Chemical and Biological Engineering Department of Chemistry Hong Kong Branch of Chinese National Engineering Research Center for Tissue Restoration and Reconstruction State Key Laboratory of Molecular Neuroscience The Hong Kong University of Science & Technology Hong Kong 999077 P. R. China; ^2^ School of Chinese Medicine Li Ka Shing Faculty of Medicine The University of Hong Kong Hong Kong 999077 P. R. China; ^3^ HKUST‐Shenzhen Research Institute No. 9 Yuexing 1st RD, South Area Hi‐Tech Park, Nanshan Shenzhen 518057 P. R. China; ^4^ Department of Chemistry The Hong Kong University of Science & Technology Hong Kong 999077 P. R. China; ^5^ The Ninth Medical Center of Chinese PLA General Hospital Beijing 100101 P. R. China; ^6^ School of Science and Engineering Shenzhen Institute of Aggregate Science and Technology The Chinese University of Hong Kong, Shenzhen Shenzhen 518172 P. R. China

**Keywords:** aggregation‐induced emission, aptamer, breast cancer, nanoparticles, photodynamic therapy

## Abstract

Breast cancer (BC), characterized by its heterogeneity and diverse subtypes, necessitates personalized treatment strategies. This study presents MF3Ec‐TBPP nanoparticles (NPs) as a promising approach, integrating an aggregation‐induced emission (AIE)‐based photosensitizer, TBPP, with the MF3Ec aptamer to enhance targeted photodynamic therapy (PDT) for Luminal A subtype BC cells. The nanoparticles also feature a 1, 2‐distearoyl‐sn‐glycero‐3‐phosphoethanolamine‐poly(ethylene glycol) shell and dipalmitoyl phosphatidylcholine (DPPC), which stabilize the structure and inhibit singlet oxygen generation, effectively reducing off‐target effects and protecting healthy tissues. Comprehensive in vitro and in vivo studies validate the NPs’ specificity and effectiveness in targeting MCF‐7 BC cells, achieving significant tumor growth inhibition with minimal damage to surrounding tissues. This study highlights the dual functionality of MF3Ec‐TBPP NPs for both diagnosis and treatment, showcasing their potential to improve patient outcomes through precise diagnostic and therapeutic interventions.

## Graphical abstract

This study presents MF3Ec‐TBPP nanoparticles for targeted photodynamic therapy of Luminal A breast cancer. These nanoparticles combine an AIE‐based photosensitizer with an aptamer. Tests show they effectively inhibit tumor growth while protecting healthy tissue via aggregation and dipalmitoylphosphatidylcholine, indicating potential for improved patient outcomes.

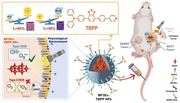

## 1 Introduction

Breast cancer (BC) represents a highly complex and heterogeneous group of diseases, necessitating personalized therapeutic strategies owing to its diverse biological nature.^[^ ^1^ ^]^ It is classified into several subtypes based on distinct biological and molecular characteristics, including Luminal A (characterized by estrogen receptor/progesterone receptor (ER/PR) positivity, HER2 negativity, and a low Ki‐67 proliferation index), Luminal B (defined by ER positivity or HER2 positivity), HER2‐enriched (noted for the amplification and overexpression of the HER2 oncogene), and triple‐negative/basal‐like (defined by the absence of ER, PR, and HER2 expressions).^[^ ^2^ ^]^ Epidemiological data from China show a significant rise in BC cases, mortality rates, and disability‐adjusted life years from 1990 to 2019. In 2019 alone, there were approximately 375 484 new cases and 96 306 deaths related to BC.^[^ ^3^ ^]^ Although advancements in early detection and the development of targeted therapies have contributed to reductions in mortality, breast cancer incidence rates continue to increase, particularly among young women.^[^ ^4^ ^]^ Therefore, there is an urgent need for the development of new therapeutic drugs and technologies to effectively treat breast cancer.

**Scheme 1:**
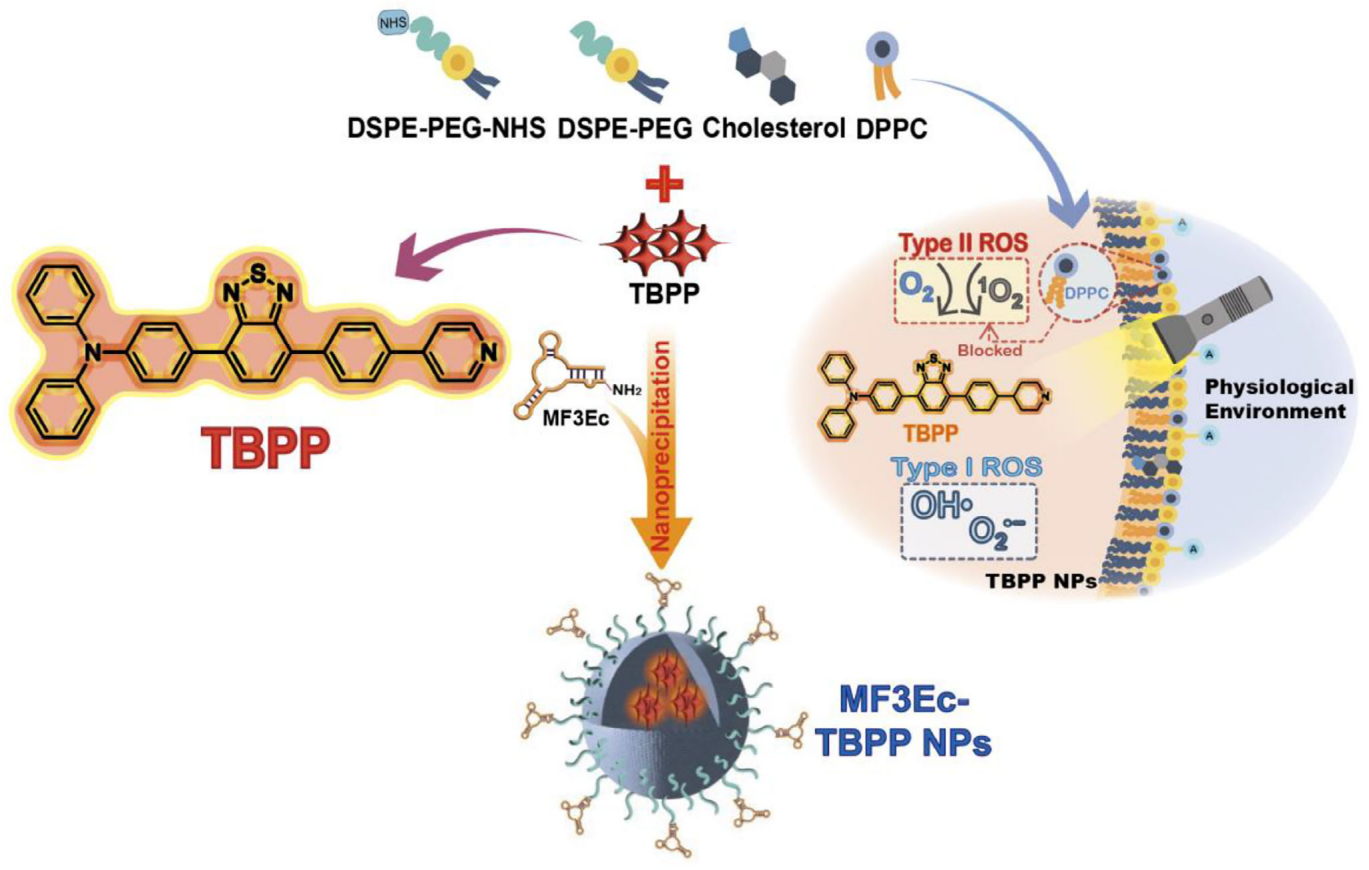
Schematic illustration of the fabrication of MF3Ec‐TBPP NPs and the ROS generation of TBPP inside the nanoparticle under light irradiation.

Traditional methods for identifying BC subtypes typically rely on histopathological examination and biomarker testing, such as immunohistochemistry and reverse transcription polymerase chain reaction.^[^ ^5^ ^]^ While these methods are cost‐effective and relatively easy to implement, they can be complex and lack accuracy, which limits their effectiveness in precision medicine. Thus, there is an urgent need to develop more accurate tools for cancer subtype identification. Aptamers are short, single‐stranded oligonucleotides, that serve as alternatives to antibodies, offering high specificity and binding affinity, stability, low production costs, ease of modification, and nonimmunogenicity.^[^ ^6^ ^]^ They have been recognized as valuable tools in the diagnosis and treatment of breast cancer as they can target specific biomarkers such as HER2 and hormone receptors, to identify different breast cancer subtypes.^[^ ^7^ ^]^ For example, aptamers have been developed to recognize subpopulations of triple‐negative breast cancer cells, which are often more challenging to diagnose and treat.^[^ ^8^ ^]^ These advancements in aptamer technology hold promise for improving the accuracy of breast cancer subtype identification and enhancing patient outcomes.

Another promising advancement in breast cancer treatment is the use of aggregation‐induced emission (AIE) materials, specifically as photosensitizers (PSs) in photodynamic therapy (PDT).^[^ ^9^ ^]^ PDT is a noninvasive therapeutic approach that utilizes light‐sensitive compounds, known as PSs, in conjunction with light of a specific wavelength to generate reactive oxygen species (ROS) that can eradicate cancer cells.^[^ ^10^ ^]^ The effectiveness of PDT is significantly influenced by the intrinsic properties of the employed PSs.^[^ ^11^ ^]^ AIE represents a unique photophysical phenomenon where certain materials exhibit enhanced luminescence upon aggregation, rather than being quenched, which is a common issue with conventional fluorophore that tend to lose their emissive capabilities in aggregation.^[^ ^12^ ^]^ The combination of AIE and PDT offers a transformative approach, wherein AIE materials maintain and even enhance their luminescent and PDT properties when aggregated.^[^ ^13^ ^]^ This characteristic enables them to deliver unique therapeutic and imaging benefits when concentrated in tumor tissues, simultaneously minimizing phototoxic effects in nontargeted, healthy tissues, thereby enhancing the precision and efficacy of therapeutic interventions.^[^ ^14^ ^]^ As a result, AIE‐based PSs hold considerable promise in optimizing PDT outcomes, offering a superior stratagem for cancer treatment refinements.

Building on these advancements, we have developed a nanoparticle platform that integrates aptamers with an AIE PS for targeted PDT of MCF‐7 cancer cells, a representative model of Luminal A subtype breast cancer (**Scheme** **1**). We utilize the AIE‐based PS TBPP, which was reported in our previous research, as the photodynamic therapeutic molecule.^[^ ^15^ ^]^ TBPP possesses several advantages that make it highly suitable for constructing the nanoparticle core. Subsequently, we developed MF3Ec‐TBPP nanoparticles (NPs) by combining the MF3Ec aptamer and a PEG shell with TBPP. These aptamer‐modified NPs demonstrate excellent optical properties and PDT efficacy. Notably, we found that dipalmitoylphosphatidylcholine (DPPC) not only stabilized the NPs structure but also effectively suppressed the generation of Type‐II singlet oxygen by TBPP, which helped minimize damage to nontarget tissues. This NP platform specifically targets and treats MCF‐7 cells in both in vitro and in vivo. Administered via tail vein injection, it enables in vivo tracing and PDT therapy in a mouse model of BC, resulting in significant tumor growth inhibition. This synergistic approach ensures specific targeting and destruction of cancer cells while minimizing harm to healthy tissues. Additionally, the strong fluorescence of AIE materials facilitates accurate imaging and real‐time monitoring of treatment efficacy. This work represents a move towards more personalized and effective treatment options, improving patient outcomes through a dual‐functional strategy that precisely diagnoses and treats cancer.

## 2 Result and Discussion

### 2.1 Photophysical Properties of TBPP Aggregates

The design of PSs with AIE characteristics is critical for enhancing their performance in cancer imaging and therapy. Building on previous studies,^[^ ^15^ ^]^ we explored the AIE‐active molecule TBPP, which consists of triphenylamine (TPA) moieties acting as electron‐donating molecular rotors and benzothiadiazole units serving as electron acceptors, thereby forming a typical donor‐acceptor (D–A) system (Figure S1, Supporting Information). This molecular design imparts TBPP with beneficial photophysical properties, including intense near‐infrared (NIR) emission, good photostability, high hydrophobicity, and low dark toxicity. Its ability to effectively generate ROS is vital for triggering cell death in cancerous tissues. Additionally, TBPP has demonstrated the ability to inhibit tumor growth in mouse models, highlighting its significant potential for clinical applications in cancer treatment.

**Figure 1:**
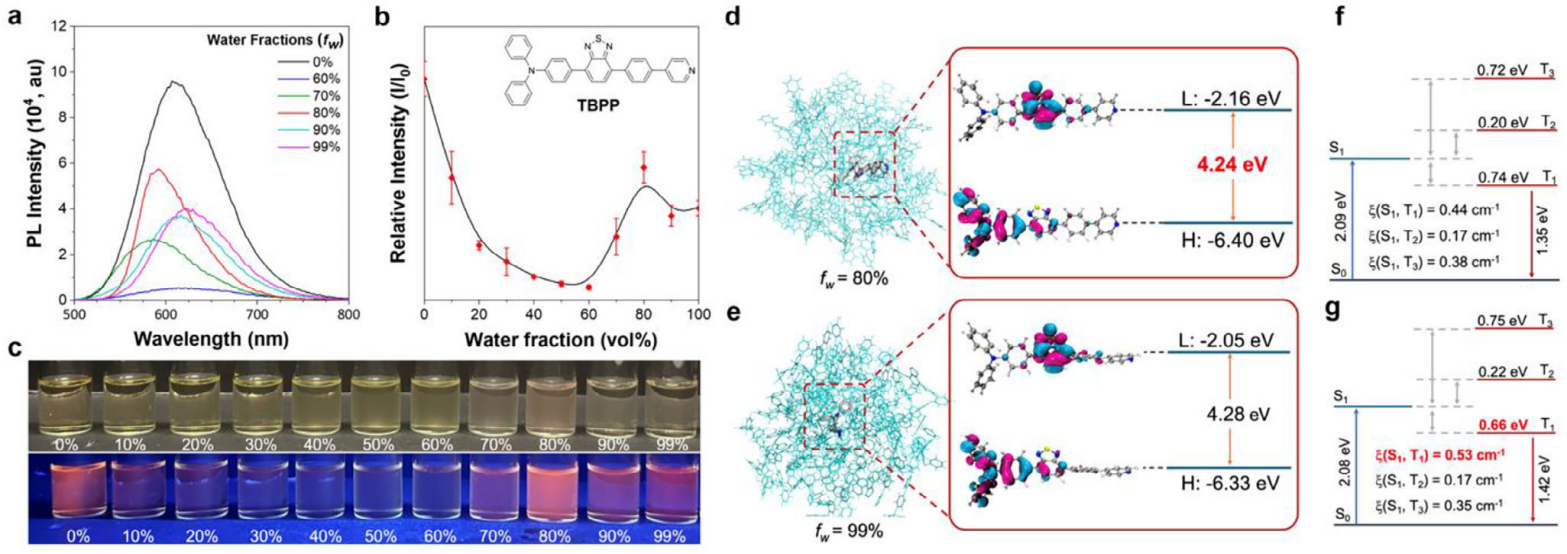
Photophysical properties of TBPP. a) Photoluminescence (PL) spectra of TBPP in water/THF mixtures with varying water fractions (*f*_W_). [TBPP] = 2 × 10^−5^ м; λ_ex_ = 460 nm. b) Plot of relative PL intensity (*I/I*_0_) of TBPP at 605 nm versus *f*_W_, where *I*_0_ represents the PL intensity of TBPP in pure THF (*f*_W_ = 0%). [TBPP] = 2 × 10^−5^ м; *λ*_ex_ = 460 nm. c) Photographs of TBPP solutions or aggregates at different water/THF mixtures with varying *f*_W_, captured under room light (top) and a 365 nm UV lamp (bottom). d,e) Molecular dynamics simulation snapshots of TBPP aggregates formed in water/THF mixture at *f*_W_ = 80% and 99%, and frontier molecular orbitals with corresponding energy levels, labeled with calculated *ΔE* in the ground state; *H*: HOMO, *L*: LUMO. f,g) Energy levels with spin‐orbit coupling (SOC) in the excited state of the innermost TBPP molecule for *f*_W_ = 80% and 99%, calculated at the M062X/6‐311G** level.

We investigated the photophysical properties of TBPP in water/tetrahydrofuran (THF) mixtures, where water acts as a poor solvent for promoting molecular aggregation. As depicted in **Figure** **1**a, TBPP demonstrates classic AIE behavior, with a pronounced increase in photoluminescence (PL) intensity observed when the water fraction (*f*_W_) surpasses 70%. These results are consistent with previously reported findings.^[^ ^15^ ^]^ Notably, we observed an unusual blue shift in the emission spectrum at *f*_W_ = 80% (Figure 1b). Additionally, the solution at *f*_W_ = 80% appears more turbid compared to that at *f*_W_ = 99% (Figure 1c and Video S1, Supporting Information). These findings suggest that the degree of aggregation of TBPP at specific water fractions leads to significant changes in its photophysical properties.

To elucidate the underlying mechanisms of these phenomena, we conducted molecular dynamics simulations to analyze the configurational changes of TBPP aggregates at *f*_W_ = 80% and *f*_W_ = 99%. (Figure 1d–g; Figures S2 and S3, Supporting Information) We validated the reliability of the simulations using root mean square deviation and solvent‐accessible surface area measurements, which showed that variations in water content significantly influence the configuration of the aggregates (Figures S2–S4, Supporting Information). To further investigate the relationship between structure and photophysical properties, we performed quantum chemical calculations on individual molecular configurations within the aggregates. The results revealed that TBPP molecules adopt distinct configurations at these water fractions, leading to differences in the energy gaps (*ΔE*) between the highest occupied molecular orbital (HOMO) and the lowest unoccupied molecular orbital (LUMO). At *f*_W_ = 80%, TBPP molecules exhibit twisted configurations of the phenylpyridine moieties relative to the TPA‐benzothiadiazole units, resulting in *ΔE* of 4.24 eV. In contrast, at *f*_W_ = 99%, a more planar configuration is observed, with an increased *ΔE* of 4.28 eV. This higher *ΔE* value impedes the excitation process, resulting in weaker emission (Figure 1d,e).

We also examined the intersystem crossing (ISC) ability of TBPP aggregates through spin‐orbit coupling (SOC) calculations. Our findings revealed multiple energy transitions from the lowest singlet excited state (S_1_) to various triplet states (T_n_). Notably, the energy gap between S_1_ and T_1_ (*ΔE*_ST_) at *f*_W_ = 80% aggregates was measured at 0.74 eV, which was larger than the 0.66 eV measured at *f*_W_ = 99%. Additionally, the results also indicate that the S_1_–T_1_ SOC coefficient (*ξ*(S_1_,T_1_)) increased to 0.53 cm^−1^ at *f*_W_ = 99%, compared to 0.44 cm^−1^ at *f*_W_ = 80%. The decrease in *ΔE*_ST_ values and the increase in *ξ*(S_1_,T_1_) at *f*_W_ = 99% suggest enhanced ISC, which may lead to diminished fluorescence and potentially enhanced ROS generation (Figure 1f,g). These findings are consistent with our experimental results, emphasizing the significant impact of molecular aggregation at different water fractions on the photophysical properties of TBPP.

### 2.2 Strategic Design and Characterization of MF3Ec‐TBPP NPs

**Figure 2:**
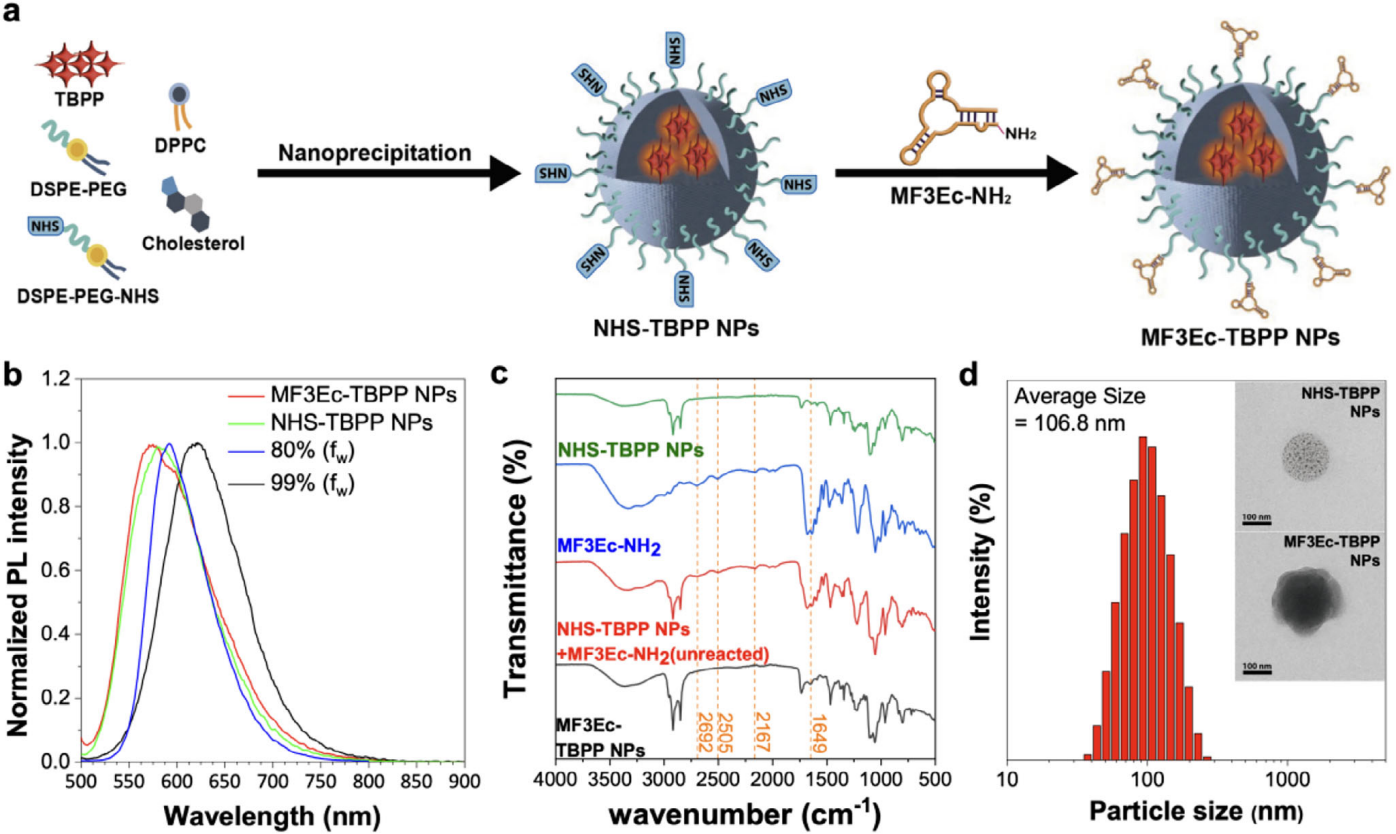
Characterization of MF3Ec‐TBPP NPs. a) Schematic representation of the fabrication process of MF3Ec‐TBPP NPs. b) Normalized photoluminescence (PL) spectra of TBPP aggregates, NHS‐TBPP NPs, and MF3Ec‐TBPP NPs. c) Fourier transform infrared spectroscopy spectra of mixtures of MF3Ec‐TBPP NPs and their components before and after reactions for surface modification. *Green*: NHS‐TBPP NPs; *Blue*: MF3Ec aptamer conjugated with anime (MF3Ec‐NH_2_); *Red:a* mixture of NHS‐TBPP NPs and MF3Ec‐NH_2_; *Black*: MF3Ec‐TBPP NPs. d) Particle size measurement of MF3Ec‐TBPP NPs by dynamic light scattering; inset: TEM images of NHS‐TBPP NPs and MF3Ec‐TBPP NPs at 40000× magnification; scale bar = 100 nm.

To achieve targeted specificity for TBPP NPs, a MF3Ec aptamer, which selectively binds to MCF‐7 BC cells, was conjugated onto the 1, 2‐distearoyl‐sn‐glycero‐3‐phosphoethanolamine‐poly(ethylene glycol) (DSPE‐PEG) NP surface. The addition of stabilizing agents such as cholesterol and lecithin (e.g., DPPC) can further enhance the stability, biocompatibility, and cellular uptake of these nanoparticles,^[^ ^16^ ^]^ resulting in the formation of MF3Ec‐TBPP NPs (**Figures** **2**a and S1, Supporting Information). The MF3Ec aptamer was purified and characterized by ATCG limited (Figure S5, Supporting Information). MCF‐7 cells, representative of the Luminal A subtype, are the most prevalent form of breast cancer, yet effective targeted therapies for this subtype are still lacking.^[^ ^17^ ^]^ Subsequently, the MF3Ec‐TBPP NPs underwent comprehensive characterization. First, UV–vis absorption measurements were conducted. The results indicate that NHS‐TBPP NPs retained an absorption spectrum similar to TBPP aggregates, suggesting that the TBPP molecules remain aggregated within the nanoparticles, thereby confirming successful encapsulation (Figure 2b). The conjugation with the MF3Ec aptamer resulted in the emergence of a distinct peak associated with the DNA aptamer, confirming successful aptamer‐nanoparticle linkage (Figure S6a, Supporting Information). Further analysis involved comparing the PL spectra of MF3Ec‐TBPP and NHS‐TBPP NPs with those of TBPP aggregates at *f*_W_ of 80% and 99% (Figure 2b). The PL spectrum of the MF3Ec‐TBPP and NHS‐TBPP NPs showed a notable blue shift, closely resembling aggregates at *f*_W_ = 80%, suggesting structural similarities that warrant further investigation in subsequent sections. Fourier‐transform infrared (FTIR) spectroscopy was employed to detect specific functional groups before and after aptamer conjugation. Comparing the FTIR spectra of NHS‐TBPP NPs, amine‐functionalized MF3Ec aptamer (MF3Ec‐NH_2_), and their mixtures pre‐ and post‐conjugation (Figure 2c), the MF3Ec‐NH_2_ displayed distinctive peaks at 2167, 2505, and 2692 cm^−1^, along with a strong peak at 1649 cm^−1^ that were absent in the spectrum of NHS‐TBPP NP. The disappearance of NH_2_ signature peaks and the appearance of new fragmental peaks indicated successful amide bond formation. Detailed FTIR spectra are provided in Figure S7 (Supporting Information). The zeta potential curves of MF3Ec‐TBPP and NHS‐TBPP NPs were measured to compare their surface charges, further confirming the successful decoration of MF3Ec aptamers on the nanoparticle surface (Figure S8, Supporting Information). Dynamic light scattering (DLS) was utilized to measure nanoparticle sizes, with NHS‐TBPP NPs and MF3Ec‐TBPP NPs showing average sizes of 103.7 and 106.8 nm, respectively (Figure 2d; Figure S6b, Supporting Information). The slight increase in particle size upon aptamer conjugation aligns with structural anticipation. Transmission electron microscopy (TEM) images further validated these findings, revealing morphological differences between NHS‐TBPP NPs and MF3Ec‐TBPP NPs. NHS‐TBPP NPs displayed a relatively rough surface with well‐defined details, whereas MF3Ec‐TBPP NPs exhibited a smoother, more diffuse surface, appearing to be coated with a transparent layer (Figure 2d; Figure S9, Supporting Information).

### 2.3 PDT Performance of TBPP Aggregates and Nanoparticles

Considering that the differences in ISC efficiency of TBPP aggregates and NPs prepared at varying water fractions can significantly impact their ROS generation, we investigated the ROS generation efficiency of TBPP NPs and aggregates specifically at 80% and 99% water fractions. We compared these findings with those of commercial PSs, including Chlorin e6 (Ce6), 2',7'‐dichlorodihydrofluorescein (DCFH), and 9,10‐anthracenediyl‐bis(methylene)dimalonic acid (ABDA).

#### 2.3.1 Evaluation of Type‐I ROS Generation

ROS are generally classified into two main types: Type‐I ROS, which involve radical species that participate in one‐electron transfer reactions (e.g., hydroxyl radicals [·OH], superoxide anions [O_2_^•−^], and peroxyl radicals [ROO·]); and Type‐II ROS, which consist of nonradical species involved in two‐electron transfer processes (e.g., hydrogen peroxide [H_2_O_2_] and singlet oxygen [^1^O_2_]). To determine the predominant ROS generated by the PSs, we employed fluorescent indicators that are specific to each type of ROS.

**Figure 3:**
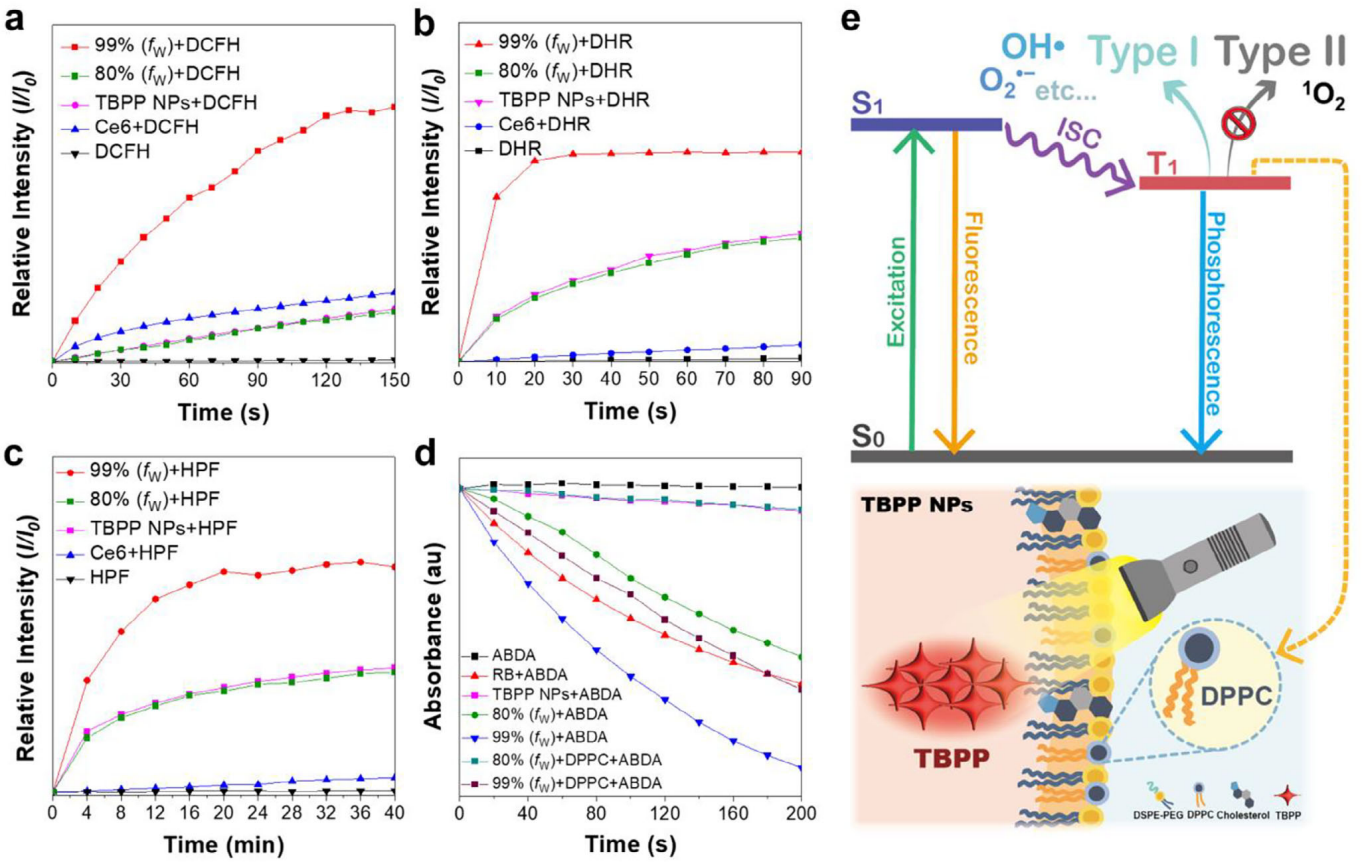
Evaluation of ROS‐generating efficiency of TBPP aggregates and NPs. a) Enhancement rate of the emission signal at 525 nm by DCFH in the presence of TBPP aggregates, TBPP NPs, and Chlorin e6 (Ce6) under white light irradiation (10 mW·cm^−^^2^). [DCFH] = 5 × 10^−5^ м, [TBPP] = [TBPP NPs] = [Ce6] = 1 × 10^−5^ м; *λ*_ex_ = 488 nm. b) Enhancement rate of emission signal at 536 nm by DHR mixed with different PSs under white light irradiation (10 mW·cm^−2^). [DHR] = 1 × 10^−5^ м; *λ*_ex_ = 505 nm. c) Enhancement rate of emission signal at 515 nm by HPF mixed with different PSs under white light irradiation (10 mW·cm^−2^). [HPF] = 1 × 10^−5^ м; *λ*_ex_ = 498 nm. d) Reduction rate of absorbance at 378 nm of ABDA in the presence of TBPP aggregates, TBPP NPs, Rose Bengal, and mixtures of aggregates and DPPC after 200 s of light irradiation. [ABDA] = [DPPC] = 1 × 10^−4^ м. These measurements were performed under white light irradiation (10 mW·cm^−2^). e) A proposed mechanism illustrating the influence of DPPC on the ROS generation of TBPP NPs.

We evaluated the total ROS generation of PSs using DCFH, a compound that reacts with ROS to produce fluorescent substance 2',7'‐dichlorofluorescein (DCF). Our analysis concentrated on bare TBPP NPs composed of DSPE‐PEG‐2000, cholesterol, DPPC, and TBPP, ensuring no interference from NHS ester groups or aptamers. For comparison, we used Ce6 as a reference PS. We observed that TBPP aggregates at *f*_W_ of 99% produced more than twice the amount of ROS compared to Ce6 (**Figure** **3**a), indicating TBPP's superior performance as an AIE‐based PS. However, its ROS production decreased significantly at *f*_W_ of 80%, falling slightly below the level observed with Ce6. This reduction aligns with our SOC calculations, which indicate a decrease in ISC efficiency at *f*_W_ = 80%. Additionally, TBPP NPs showed similar ROS generation to the *f*_W_ = 80% aggregates, indicating that the molecular aggregates within the NPs mimic the behavior of those at 80% *f*_W_.

In addition to measuring total ROS, we also examined the specific production of Type‐I ROS by TBPP aggregates and NPs. The generation of superoxide anion (O_2_^•−^) was evaluated with dihydrorhodamine 123 (DHR123). The results showed that TBPP aggregates prepared at *f*_W_ of 99% had the highest superoxide output while the TBPP aggregates formed *f*_W_ = 80% and TBPP NPs produced moderately elevated levels of O_2_^•−^ (Figure 3b), consistent with the DCFH results. For hydroxyl radical (OH·) production, we used hydroxyphenyl fluorescein (HPF) to assess (Figure 3c). Again, TBPP aggregates (*f*_W_ = 99%) led in generation, although at a slower rate compared to the superoxide. Both TBPP aggregates (*f*_W_ = 80%) and TBPP NPs showed similar moderate increases in emission. In contrast, Ce6 displayed minimal change in emission, reflecting its established inefficiency in hydroxyl radical production.

#### 2.3.2 Evaluation of Type‐II ROS Generation

To measure singlet oxygen production by TBPP, we used ABDA, which detects singlet oxygen through a decrease in absorbance. We evaluated TBPP aggregates prepared at *f*_W_ of 99% and 80%, TBPP NPs, and the commercial PS, Rose Bengal (RB) (Figure 3d). TBPP aggregates (*f*_W_ = 99%) yielded the highest levels of singlet oxygen. In contrast, the aggregates (*f*_W_ = 80%) and TBPP NPs generated considerably less singlet oxygen, with TBPP NPs showing even lower production compared to the TBPP aggregates (*f*_W_ = 80%). This finding was not expected, as we initially hypothesized that the TBPP NPs would mimic the aggregates (*f*_W_ = 80%). The notable reduction in singlet oxygen suggests that other factors may affect the PDT performance of TBPP NPs.

To investigate the cause of the reduction in singlet oxygen generation observed in TBPP NPs, we carefully examined the roles of two key components in the NPs, cholesterol and DPPC. These components were selected because they are critical structural elements of the nanoparticle formulation, with cholesterol modulating membrane fluidity and DPPC forming the primary lipid matrix. Their influence on the photophysical behavior of TBPP was hypothesized to play a pivotal role in singlet oxygen generation. By adding each component individually to solutions containing TBPP aggregates (*f*_W_ = 99%), we found that DPPC caused a more than 20% reduction in singlet oxygen production, while cholesterol showed no significant effect (Figure S10a, Supporting Information). This indicates that DPPC is primarily responsible for the decreased singlet oxygen generation in TBPP NPs compared to the TBPP aggregates (*f*_W_ = 80%). To further validate the role of DPPC, we repeated ABDA assays with TBPP aggregates (*f*_W_ = 80%) mixing with DPPC, and TBPP NPs (Figure 3d). The singlet oxygen levels in the aggregates mixed with DPPC matched those observed in the NPs, clearly demonstrating the impart of DPPC on singlet oxygen reduction in the NP context.

To further investigate the photophysical mechanism underlying this reduction, we conducted transient absorption (TA) spectroscopy to analyze the decay dynamics of photoinduced excited states. As shown in Figure S11 (Supporting Information), the TA spectra of TBPP aggregates (*f*_W_ = 99%) in the absence and presence of DPPC revealed a negative ground state bleach (GSB) below 550 nm and a broad singlet excited state absorption (ESA) band at ≈700 nm. Additionally, a transient positive band at ≈575 nm, attributed to triplet ESA, was observed. However, upon the addition of DPPC, the triplet ESA signal underwent a twofold reduction in amplitude and decayed completely within 1 ns, compared to the pristine TBPP aggregates, where 25% of the signal persisted for up to 7 ns (Figure S12, Supporting Information). These results suggest that DPPC introduces a competitive quenching pathway for the triplet excited state, thereby reducing singlet oxygen generation. This reduction in singlet oxygen generation not only limits off‐target effects in photodynamic therapy by reducing singlet oxygen diffusion to healthy surrounding tissues but also enhances control over the therapeutic process.

Furthermore, using DCFH, DHR123, and HPF indicators, we observed that DPPC did not significantly affect the generation of other types of ROS (Figures S13−S16, Supporting Information). These results suggest that our strategic design of TBPP NPs can effectively reduce singlet oxygen generation during transit, thereby minimizing premature ROS generation that could damage nontarget tissues. The presence of DPPC in the NP formulation further decreases singlet oxygen generation, thus minimizing off‐target effects. Additionally, we conducted a supplementary study using ^31^P NMR and TBPP emission measurements to determine whether DPPC undergoes structural changes after prolonged irradiation with TBPP, or if it directly reacts with TBPP. The absence of significant ^31^P peak shifts and no changes in TBPP emission wavelengths indicated that DPPC neither chemically reacts with singlet oxygen nor alters the TBPP structure (Figure S17, Supporting Information). In short summary, our findings confirm that the ROS generation performance of TBPP NPs closely resembles to that of the TBPP aggregates (*f*_W_ = 80%). This indicates that the PDT activity of the encapsulated TBPP molecule may be similar to that of the aggregates but could exhibit a controlled reduction. Furthermore, the introduction of DPPC further limits singlet oxygen production, offering notable advantages by reducing off‐target PDT effects and enhancing control over the PDT process without significantly compromising therapeutic efficacy (Figure 3e).

### 2.4 In Vitro Imaging and Targeted PDT of MCF‐7 Cells

#### 2.4.1 In Vitro Imaging of MCF‐7 Cells by MF3Ec‐TBPP NPs

After characterizing MF3Ec‐TBPP NPs and evaluating their ROS performance, we proceeded to investigate their efficacy for targeted PDT in breast cancer cells. We first examined the distribution of MF3Ec‐TBPP NPs in breast cancer cells using confocal laser scanning microscopy (CLSM). Previous studies showed that FAM‐labeled MF3Ec selectively binds to the cell membrane of MCF‐7 cells, but not to SK‐BR‐3 (HER2‐positive), MDA‐MB‐231 (triple‐negative), or MCF‐10A (human breast epithelial) cells.^[^ ^18^ ^]^ Based on these findings, we hypothesized that MF3Ec‐TBPP NPs would similarly bind to the membrane of MCF‐7 cells.

**Figure 4:**
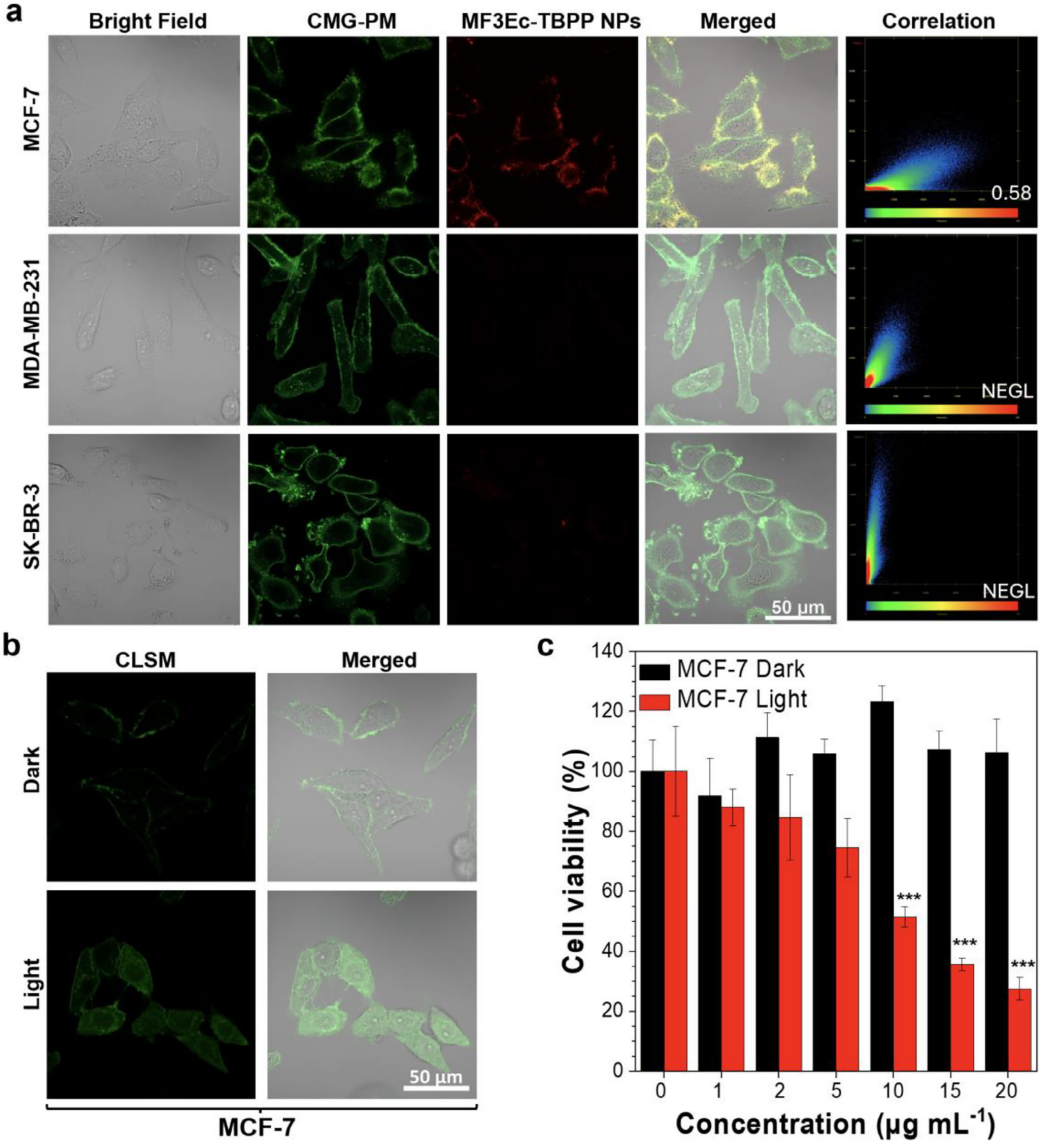
In vitro imaging and PDT evaluation of MF3Ec‐TBPP NPs. a) Bright‐field and CLSM images of MCF‐7, SK‐BR‐3, and MDA‐MB‐231 cells incubated with MF3Ec‐TBPP NPs (10 µg·mL^−1^) and CellMask^TM^ green plasma membrane stain (CMG‐PM, 1×) for 15 min. Objective lens = 40×; scale bar = 50 µm; *λ*_ex_ = 488 nm; MF3Ec‐TBPP NPs: *λ*_em_ = 600−700 nm; CMG‐PM: *λ*_em_ = 500−550 nm. b) Bright‐field and CLSM images of MCF‐7 cells treated with DCFH (1 × 10^−2^ м) and incubated with MF3Ec‐TBPP NPs (10 µg·mL^−1^) in a dark environment or under white light irradiation (20 mW·cm^−2^) for 30 min. *λ*_ex_ = 488 nm; *λ*_em_ = 500−550 nm; objective lens = 40×; scale bar = 50 µm. c) MTT assay for MCF‐7 cells incubated with MF3Ec‐TBPP NPs in a dark environment or under white light irradiation (20 mW·cm^−2^) in DMEM medium for 40 min. All data shown are expressed as mean values ± standard deviation with a sample size of *n* = 7; *p < 0.5, **p < 0.01, and ***p < 0.001.

To validate our hypothesis, we co‐stained different cancer cells with MF3Ec‐TBPP NPs and CellMask™ plasma membrane stains (CMG‐PM) to determine whether the NPs localized on the cell surface (Figure S18, Supporting Information; **Figure** **4**a). As expected, red fluorescence predominantly observed at the surface of MCF‐7 cells, with negligible signals detected in negative control cells (BT‐474, SK‐BR‐3, and MDA‐MB‐231). These results were consistent with the targeting results from co‐culturing MCF‐7 and SK‐BR‐3 cells (Figure S19, Supporting Information), and indicate that MF3Ec‐TBPP NPs effectively target MCF‐7 cells. The Pearson's correlation coefficient between MF3Ec‐TBPP NPs and CMG‐PM in MCF‐7 cells was 0.58, indicating partial overlap of the fluorescent signals. This degree of colocalization aligns with our hypothesis, as existing literature suggests that the MF3Ec aptamer targets the cytomembrane protein PHB2, whose specific identity and distribution remain under investigation.^[^ ^18b^ ^]^ It is plausible that this protein is localized to distinct regions of the membrane rather than evenly distributed across the surface, which could explain the observed Pearson's correlation coefficient and the expected localization pattern.

#### 2.4.2 Monitoring the Release and the Intracellular ROS Generation of TBPP

To thoroughly investigate the release dynamics of TBPP aggregates from MF3Ec‐TBPP NPs, we employed dialysis to quantify the release, revealing that 30% of TBPP aggregates were liberated within the first hour (Figure S20, Supporting Information). We also complemented this analysis with time‐lapse cell imaging. We stained MCF‐7 cells with MF3Ec‐TBPP NPs and captured the cell images at various intervals. As illustrated in Figure S21 (Supporting Information), TBPP release commenced after 30 minutes, with the red fluorescent signal shifting from the plasma membrane to the interior of MCF‐7 cells. This observation aligns with previous findings that TBPP aggregates are internalized and accumulated within lipid droplets.^[^ ^15^ ^]^ These results suggest that the inactivation of PDT in TBPP NPs does not compromise therapeutic efficacy, as the TBPP molecules are released and taken up by target cells in aggregate form, where they become reactivated upon aggregation to generate ROS at the tumor site (Figure S22, Supporting Information).

We further investigated the ROS production within cells using DCFH‐DA as a fluorescent probe. MCF‐7 or SK‐BR‐3 cells were incubated with DCFH‐DA and MF3Ec‐TBPP NPs, followed by exposure to white light irradiation (20 mW·cm^−2^) for 30 min (Figure 4b; Figure S23a, Supporting Information). The irradiated MCF‐7 cells exhibited strong green fluorescence, indicating effective intracellular ROS generation by TBPP. In contrast, MCF‐7 cells kept in the dark and SK‐BR‐3 cells displayed minimal fluorescence. We expanded our study to include hypoxia conditions, where MCF‐7 cells treated with MF3Ec‐TBPP NPs exhibited weakened fluorescence after irradiation compared to normal conditions, but slightly enhanced fluorescence over dark controls. These results suggest that oxygen plays a critical role in ROS generation, consistent with Type‐II ROS production (Figure S24, Supporting Information). Additionally, hypoxia‐induced morphological changes, including cell shrinkage and intracellular bubbles, may contribute to reduced nanoparticle uptake and ROS generation efficiency.

To evaluate the in vitro PDT performance of MF3Ec‐TBPP NPs on MCF‐7 cells, we employed the MTT assay to assess both phototoxicity and dark toxicity. SK‐BR‐3 cells served as a negative control (Figure S23b, Supporting Information). After PDT, we observed a significant reduction in cell viability in the light‐irradiated MCF‐7 cells, with cell death proportional to the concentration of MF3Ec‐TBPP NPs. In contrast, the dark control groups for both cell lines showed negligible cell death, confirming that MF3Ec‐TBPP NPs do not exhibit any observable cytotoxicity in the absence of light (Figure 4c). To further evaluate the phototoxicity of MF3Ec‐TBPP NPs, we plotted the concentration of MF3Ec‐TBPP NPs against cell viability in MCF‐7 cells following light irradiation. The half‐maximal inhibitory concentration (IC_50_) was calculated to be 11.2 µg·mL^−1^, as determined by linear regression analysis (Figure S25, Supporting Information). The observed phototoxicity in SK‐BR‐3 cells is likely caused by trace amounts of TBPP molecules diffusing into the cells at higher concentrations, combined with their higher susceptibility to ROS. However, this toxicity is still less prominent compared to that observed in MCF‐7 cells.

In short summary, these findings demonstrate that MF3Ec‐TBPP NPs are highly effective for targeted PDT in MCF‐7 cells, inducing significant cell death upon light activation while exhibiting minimal toxicity in the absence of light and in nontarget SK‐BR‐3 cells.

### 2.5 In Vivo Imaging and PDT Evaluation of MF3Ec‐TBPP NPs

**Figure 5:**
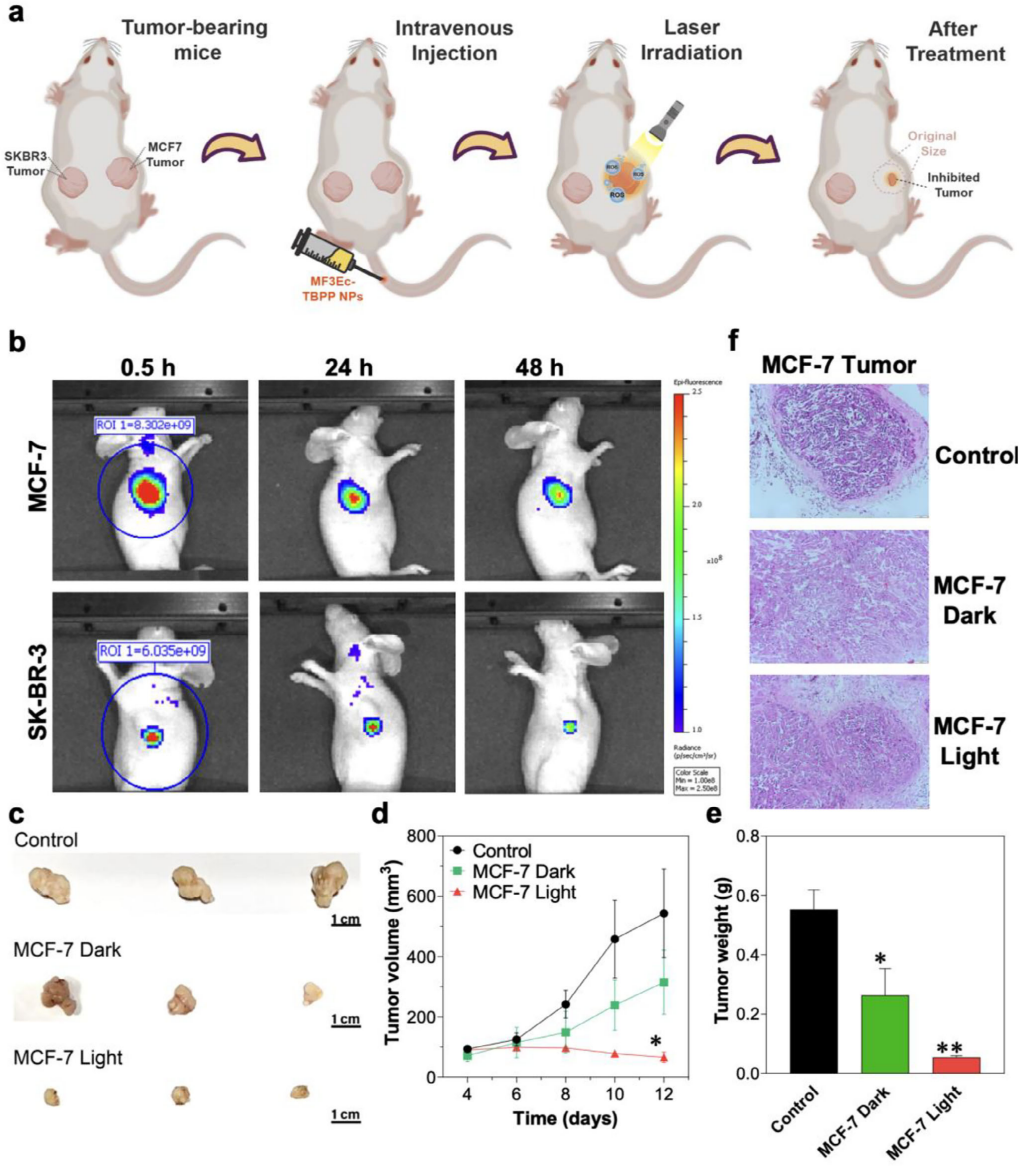
In vivo imaging and PDT evaluation in a mouse tumor model with two different subtypes of breast cancer. a) Schematic diagram illustrating fluorescent imaging and PDT of tumor‐bearing animal model. b) NIR imaging of a tumor‐bearing mouse over a 48‐h period following intravenous injection of 100 µL of a 0.5 mg·mL^−1^ MF3Ec‐TBPP NPs solution via tail vein (Each group, *n* = 3). *λ*_ex_ = 500 nm; *λ*_em_ = 700 nm. c) Representative images of tumors in mice post‐treatment with a 0.5 mg·mL^−1^ MF3Ec‐TBPP NPs solution (Each group, *n* = 3). The terms "dark" and "light" indicate whether the NP‐treated groups were not exposed or were exposed to white light (100 mW·cm^−2^ for 30 min), respectively; scale bar = 1 cm. d) Tumor volume progression in mice recorded at various time points post‐NP administration. e) Measurement of tumor mass in mice 12 days post‐NP treatment (Each group, *n* = 3). f) Hematoxylin and eosin staining of tumor sections from different treatment groups 12 days after NP treatment (Each group, *n* = 3). All data shown are expressed as mean values ± standard deviation; *p < 0.5, **p < 0.01, and ***p < 0.001.

To assess the efficacy of MF3Ec‐TBPP NPs for targeted PDT in breast cancer, we conducted in vivo experiments using a mouse model with MCF‐7 and SKBR3 tumors. The experimental design is illustrated in **Figure** **5**a, which outlines the process from tumor‐bearing mice to post‐treatment analysis. Initially, we verified the excellent photostability of MF3Ec‐TBPP NPs (Figure S26, Supporting Information). Following this, we examined how different doses of MF3Ec‐TBPP NPs solutions affected tracer efficacy by intravenously injecting different volumes of MF3Ec‐TBPP NP solutions (40, 80, and 120 µL at a concentration of 0.5 mg·mL^−1^) into the tumor‐bearing mice. Results indicated that dosages ranging from 80 to 120 µL provided excellent imaging in MCF‐7 tumors, with a clear fluorescent difference between MCF‐7 and SKBR3 tumors (Figure S27, Supporting Information). Subsequently, we used NIR imaging to monitor the distribution and concentration of MF3Ec‐TBPP NPs in the tumors. As illustrated in Figure 5b, strong fluorescent signals were detected in the MCF‐7 tumors from 0.5 to 48 h post‐injection, indicating effective targeting and accumulation of the NPs. In contrast, SKBR3 tumors exhibited minimal fluorescence and scant tracer signal coverage on the tumor area, highlighting the specificity of the MF3Ec aptamer for MCF‐7 cells. This selective targeting is vital for minimizing off‐target effects and enhancing therapeutic efficacy. Based on these promising results, we proceeded with PDT at the MCF‐7 site. The therapeutic efficacy of MF3Ec‐TBPP NPs was further evaluated by measuring tumor volume and weight. Representative images of excised tumors from various treatment groups are shown in Figure 5c. The control group exhibited substantial tumor growth, whereas the MCF‐7 Dark group (NPs without light exposure) showed potential tumor growth inhibition. In contrast, the MCF‐7 Light group (NPs with light exposure) demonstrated a significant reduction in tumor size. To validate these findings, we increased the mouse cohort to *n* = 12, with tumor images and weight changes further supporting the therapeutic efficacy of light‐activated PDT (Figure S28, Supporting Information). Quantitative analysis of tumor volume over time (Figure 5d) revealed that the MCF‐7 Light group exhibited a markedly slower tumor growth rate compared to the control and MCF‐7 Dark groups. By day 12 post‐treatment, the tumor volume in the MCF‐7 Light group was significantly smaller, highlighting the potent PDT effect of MF3Ec‐TBPP NPs under light activation. Tumor weight measurements (Figure 5e) supported these findings, with the MCF‐7 Light group showing the lowest tumor mass, followed by the MCF‐7 Dark group, and the control group having the highest tumor mass. Statistical analysis confirmed significant differences between the groups, emphasizing the enhanced therapeutic outcomes achieved with light‐activated PDT.

Subsequently, histopathological analysis of tumor tissues was performed using hematoxylin and eosin (H&E) staining to assess cellular morphology and treatment‐induced damage (Figure 5f). The tumors in the control group displayed dense cellularity with minimal necrosis, indicative of aggressive tumor growth. Conversely, the MCF‐7 Dark group showed areas of necrosis and reduced cellular density, suggesting partial therapeutic effects of the NPs even without light activation. Notably, the MCF‐7 Light group exhibited extensive necrosis and disrupted tumor architecture, consistent with effective PDT‐induced cell death. These histological findings align with the tumor volume and weight data, further validating the superior efficacy of MF3Ec‐TBPP NPs when activated by light.

Finally, an evaluation of MF3Ec‐TBPP NPs' toxicity was conducted. Over the 12‐day study period, there were no significant differences in body weight among the groups, with all maintaining normal growth patterns (Figure S29, Supporting Information). Additionally, H&E staining of major organs (heart, liver, spleen, lungs, kidneys) indicated normal tissue morphology without apparent damage (Figure S30, Supporting Information). Hemolysis assays were also performed to evaluate the blood compatibility of MF3Ec‐TBPP NPs, showing negligible hemolytic activity and confirming their excellent biosafety (Figure S31, Supporting Information). Ex vivo fluorescence imaging of organs and tumors, obtained 48 h after tail vein injection of NPs, showed that MCF‐7 tumors sustained robust fluorescent signals, while SKBR3 exhibited only minimal fluorescence. This further confirm the NPs' specificity for MCF‐7 and their good retention within the tumor (Figure S32, Supporting Information). Moreover, significant fluorescence was observed in the liver and kidneys, suggesting possible accumulation of the NPs in these organs, in line with previous reports of NP accumulation in the liver.^[^ ^19^ ^]^ This implies that the NPs may be metabolized through the kidneys and liver.

Our results demonstrate several significant advantages of the MF3Ec‐TBPP NPs. First, the MF3Ec aptamer conferred high specificity to MCF‐7 cells, which is crucial for minimizing off‐target effects and enhancing therapeutic efficacy. Second, the MF3Ec‐TBPP NPs exhibited potent PDT effects upon light activation. Third, the introduction of TBPP, an AIE‐based PS, enabled strong fluorescence for imaging and monitoring treatment efficacy. Additionally, the use of DPPC within the NP formulation offered dual functionalities. DPPC not only acted as a structural stabilizer, enhancing the integrity and stability of the NPs, but also served as a singlet oxygen inhibitor in nontarget tissues. To the best of our knowledge, this is the first report of DPPC serving such a dual role in nanoparticle‐based PDT systems. Finally, MF3Ec‐TBPP NPs are well‐tolerated in vivo and do not elicit significant systemic toxicity.

Despite these promising findings, there are limitations to our study that warrant further investigation. One notable observation was the partial suppression of tumor growth in the MCF‐7 Dark group, which received NPs without light exposure. This suggests that the NPs may exert some therapeutic effects independent of light activation, possibly due to the intrinsic cytotoxicity of the NPs or other mechanisms unrelated to PDT. Further studies are needed to elucidate these effects and optimize the formulation to minimize any unintended cytotoxicity. Another limitation is the accumulation of NPs in the liver and kidneys, which raises concerns about potential long‐term toxicity and off‐target effects. Comprehensive pharmacokinetic and biodistribution studies, along with assessments of long‐term safety and clearance mechanisms, are necessary to fully evaluate the clinical applicability of MF3Ec‐TBPP NPs. Lastly, the reliance on external light sources for PDT limits the effectiveness of this approach for treating deeply seated tumors where light penetration is insufficient. The depth of light penetration in biological tissues is a significant challenge for PDT, and strategies to enhance light delivery, such as the use of upconversion nanoparticles or implantable light sources, may need to be explored to expand the applicability of this therapy.

## 3 Conclusion

In conclusion, the MF3Ec‐TBPP nanoparticle platform presents a sophisticated approach to targeted breast cancer therapy, effectively integrating specific tumor targeting, PDT, and real‐time imaging capabilities. The integration of the MF3Ec aptamer and the AIE PS, TBPP within a DPPC and PEG shell results in a nanostructure that effectively discriminates between cancerous and noncancerous tissues, thereby minimizing off‐target effects and providing a safe yet potent therapeutic option. Addressing the limitations identified will be crucial for advancing this technology toward clinical application and unlocking its full potential in personalized cancer treatment.

## 4 Experimental Section

### Materials and Syntheses

All materials and synthetic methods are described in the Supporting Information.

### Spectrophotometric Experiments

A stock solution of TBPP in THF (1 × 10^−3^ м) was prepared and then diluted with the relevant solvents or water to reach a final dye concentration of 1 × 10^−2^ м for both absorption and PL measurements. Absorption spectra were recorded between 250 and 600 nm, while fluorescence spectra (500−900 nm) were obtained with an excitation wavelength of 450 nm. For the stock solutions of TBPP NPs, bare TBPP NPs, NHS‐TBPP NPs, and MF3Ec‐TBPP NPs were prepared following the method outlined in the Supporting Information and subsequently diluted to a concentration of 1 × 10^−2^ м (5.327 µg·mL^−1^) using DNase‐free distilled water.

### Computational Details—Aqueous Aggregate Molecular Dynamic (MD) Simulation

TBPP was optimized with the density functional theory (DFT) method using M062X‐GD3 density functional and 6‐31G(d,p) basis set in water and vacuum. The topology file was generated by Sobtop with REST2(0.5) charge calculating via Equation (1).^[^ ^20^ ^]^

(1) $$q_{REST2\left(0.5\right)}=0.5\times q_{gas}+0.5\times q_{solv}$$

The molecular dynamics simulations were performed with the GROMACS simulation package (version 4.6.5 or newer).^[^ ^21^ ^]^ The time step was set to 2 fs. The temperature was set to 298 K for each individual simulation. For temperature and pressure coupling, the v‐rescale and Parinello–Rahman algorithms were used.^[^ ^22^ ^]^ The pressure was isotropic and set to 1 bar with a water compressibility of 45 mbar^−1^. All simulations were performed with periodic boundary conditions in all three directions. The cutoff of nonbonded interactions was set to 1.0 nm. The particle mesh Ewald method was used for the long‐range electrostatic interactions.^[^ ^23^ ^]^ The force field GAFF was used.^[^ ^24^ ^]^ 46 TBPP and 335 THF molecules were placed in a 4.5 nm × 4.5 nm × 4.5 nm box followed by placing the small box into an 8 nm × 8 nm × 8 nm with 5584 SCP water molecules to simulate TBPP in THF/water mixture (*f*_w_ = 80%). 46 TBPP and 11 THF molecules were placed in a 4.5 nm × 4.5 nm × 4.5 nm box followed by placing the small box into a 8 nm × 8 nm × 8 nm with 5584 SCP water molecules to simulate TBPP in THF/water mixture (*f*_w_ = 99%). To equilibrate the system, the initial energy minimization of the system was followed by a 100 ns NPT simulation (P = 1 atm and T = 300 K). The equilibrated conformation was then adopted to perform a 300 ns production run in NPT ensemble (P = 1 atm and T = 300 K). The simulation trajectory was then used to study the behaviors of the aggregates. The innermost molecule was detailly investigated, which was defined as the molecule closest to the center of the aggregate averaged over the whole trajectory. The TBPP innermost molecule in THF/water mixture (*f*_W_ = 80% and 99%) aggregate was fully optimized with the density functional theory (DFT) method using M062X‐GD3 density functional and 6‐311G(d,p) basis set.^[^ ^25^ ^]^ To better simulate the usage scenarios (in water solution or living cells) of molecules, water with the solvation model based on density (SMD) was added to consider the bulky solvation effects in the self‐consistent reaction field (SCRF).^[^ ^26^ ^]^ Analytical frequency calculations were also performed at the same level of theory to confirm that the optimized structures were at a minimum point. Time‐dependent density functional theory (TD‐DFT) was utilized at the same level of theory to calculate energy levels of singlet, triplet states, and their gap (*ΔE_st_*).^[^ ^27^ ^]^ The above quantum chemical calculations were carried out at Gaussian 16 program.^[^ ^28^ ^]^ Besides, to evaluate the intersystem crossing efficiency of two molecules, the spin‐orbital coupling (SOC) constants were calculated based on optimized excited state geometries. The electron cloud distribution maps were treated via Multifwn software and displayed using VMD software.^[^ ^29^ ^]^

### Total ROS Generation

To prepare DCFH, 78.2 µL of DCFH‐DA (1 × 10^−3^ м in DMSO) was mixed with 421.8 µL of DMSO and 2 mL of NaOH (1 × 10^−4^ м in water) and incubated at room temperature for 30 min. Afterward, 10 mL of 1× PBS was added to dilute the solution. A mixture of 750 µL of 5 × 10^−1^ м DCFH and 10 µm of either TBPP (*f*_W_ = 99%), TBPP (*f*_w_ = 80%), Ce6 or bare TBPP‐NPs (5.33 µg·mL^−1^) was prepared, then irradiated under white light. Emission spectra were recorded every 10 s using an excitation wavelength of 488 nm.

### Superoxide Anion (O_2_^•−^) Detection

For superoxide anion detection, 10 µm DHR was mixed with 10 µm of either TBPP (*f*_W_ = 99% or *f*_W_ = 80%), bare TBPP‐NPs or Ce6 in 1× PBS. The solution was irradiated under white light, and the emission spectra were recorded every 10 s with an excitation wavelength of 505 nm.

### Hydroxyl Radical (OH•) Detection

To detect hydroxyl radicals, 1 × 10^−5^ м HPF was added to a solution containing 1 × 10^−5^ м of either TBPP (*f*_w_ = 99% or *f*_w_ = 80%), bare TBPP‐NPs or Ce6 in 1× PBS. The solution was irradiated with white light, and the emission spectra were collected every 4 min with an excitation wavelength of 498 nm.

### Singlet Oxygen (^1^O_2_) Detection

For singlet oxygen detection, 1 × 10^−4^ м ABDA was mixed with 1 × 10^−5^ м of either TBPP (*f*_W_ = 99% or *f*_W_ = 80%), bare TBPP NPs, or Rose Bengal (RB) in 1× PBS. To evaluate the effects of DPPC, 1 × 10^−4^ м DPPC or 50 µm cholesterol was combined with 1 × 10^−5^ м TBPP (*f*_W_ = 99% or *f*_W_ = 80%) and 1 × 10^−4^ м ABDA in 1× PBS. The mixtures were irradiated with white light, and absorption spectra were recorded every 2 min to monitor the reaction.

### Effects of DPPC on ^1^O_2_ generation

To determine the effects of DPPC on the ^1^O_2_ generation, different amount of DPPC was mixed with 1 × 10^−5^ м of TBPP (*f*_W_ = 99%) and 1 × 10^−4^ м ABDA. The solution was irradiated under white light for 200 s, and the absorption spectra were recorded from 350 to 410 nm. The dark control was the absorption spectra recorded by 1 × 10^−4^ м of DPPC mixed with 1 × 10^−5^ м of TBPP (*f*_W_ = 99%) and 1 × 10^−4^ м ABDA before light irradiation.

### Effects of DPPC on Total ROS Generation

To determine the effects of DPPC on the total ROS generation, different amount of DPPC was mixed with 1 × 10^−5^ м of TBPP (*f*_W_ = 99%) and 5 × 10^−5^ м DCFH. The solution was irradiated under white light for 200 s, and the PL spectra were recorded from 500 to 600 nm using an excitation wavelength of 488 nm. The dark control was the PL spectra recorded by 1 × 10^−4^ м of DPPC mixed with 10 µm of TBPP (*f*_W_ = 99%) and 5 × 10^−5^ м DCFH before light irradiation.

### Effects of DPPC on O_2_^•−^ Generation

To determine the effects of DPPC on the O_2_^•−^ generation, different amount of DPPC of DPPC was mixed with 1 × 10^−5^ м of TBPP (*f*_W_ = 99%) and 1 × 10^−5^ м DHR. The solution was irradiated under white light for 200 s, and the PL spectra were recorded from 500 to 600 nm using an excitation wavelength of 505 nm. The dark control was the PL spectra recorded by 1 × 10^−4^ м of DPPC mixed with 1 × 10^−5^ м of TBPP (*f*_W_ = 99%) and 1 × 10^−5^ м DHR before light irradiation.

### Effects of DPPC on OH• Detection

To determine the effects of DPPC on the OH• generation, different amount of DPPC was mixed with 1 × 10^−5^ м of TBPP (*f*_W_ = 99%) and 1 × 10^−5^ м HPF. The solution was irradiated under white light for 200 s, and the PL spectra were recorded from 500 to 600 nm using an excitation wavelength of 505 nm. The dark control was the PL spectra recorded by 1 × 10^−4^ м of DPPC mixed with 1 × 10^−5^ м of TBPP (*f*_W_ = 99%) and 1 × 10^−5^ м HPF before light irradiation.

### Release of TBPP from MF3Ec‐TBPP NPs (Dialysis Method)

The release behavior of TBPP from MF3Ec‐TBPP NPs was investigated using a dialysis method at 37°C. A 10 mL solution of MF3Ec‐TBPP NPs (0.1 mg·mL^−1^) was placed in dialysis bags with a molecular weight cutoff of 3000 Da and immersed in deionized water, which was stirred continuously for 24 h. At specific time intervals, 3 mL of the surrounding deionized water was removed and analyzed to assess the release of TBPP. An equal volume of fresh deionized water was added to maintain a constant total volume. The release of TBPP was quantified by measuring the absorbance of the respective solutions at 460 nm.

### Release of TBPP from MF3Ec‐TBPP NPs In Vitro

MCF‐7 cells were seeded onto glass‐bottom confocal dishes and incubated overnight to ensure proper attachment. Live‐cell imaging was then initiated using fluorescence microscopy, with the cells maintained in a humidified environment at 37 °C and 5% CO_2_ throughout the experiment. After staining with MF3Ec‐TBPP NPs (10 µg·mL^−1^) and CellMask™ Green Plasma Membrane Stain (CMG‐PM, 1×), the cells were gently washed twice with PBS. Fluorescence microscopy (LSM800, Zeiss, Germany) was employed to capture images at 10‐min intervals over a period of 60 min. The green channel for CMG‐PM was set with excitation at 488 nm and emission between 505 and 525 nm, while the TBPP channel for MF3Ec‐TBPP NPs was set with excitation at 488 nm and emission between 605 and 700 nm. The resulting imaging data were processed and analyzed using ZEN software (Zeiss, Germany).

### Cell Culture

Dulbecco's Modified Eagle Medium (DMEM), fetal bovine serum (FBS), and penicillin‐streptomycin were obtained from Thermo Fisher. Media were supplemented with 10% FBS and penicillin‐streptomycin. Cells were cultured at 37°C in a 5% CO_2_ humidified incubator.

### Cell Imaging for Evaluating Target Specificity

MCF‐7, MDA‐MB‐231, and SK‐BR‐3 live cells were incubated with MF3Ec‐TBPP NPs (10 µg·mL^−1^) and CMG‐PM (1×) for 15 min. After washing, fluorescence images were captured using an LSM800 confocal microscope with 488 nm excitation at 2% laser power. Emission was collected in the 500–525 nm range for CMG‐PM and 605−700 nm for MF3Ec‐TBPP NPs. The images were acquired using a 40× objective lens.

### Cocultured Cell Imaging

MCF‐7 and SK‐BR‐3 cells were seeded into the same culture dish with 1:1 ratio of counts in DMEM supplemented with 10% FBS and 1% penicillin‐streptomycin. The cells were cultured at 37°C in a 5% CO_2_ humidified incubator for 24 h. The cells were then stained by MF3Ec‐TBPP NPs (10 µg·mL^−1^) for 15 min and followed by washing with PBS. After washing, fluorescence images were captured using an LSM800 confocal microscope with 488 nm excitation at 2% laser power. Emission was collected in 605−700 nm for MF3Ec‐TBPP NPs. The images were acquired using a 40× objective lens.

### Determination of Total ROS Generation In Vitro

MCF‐7 and SK‐BR‐3 cells were first incubated with DCFH‐DA (20 µm) at 37 °C for 30 min in DMEM with 10% FBS and 1% PS. After washing with PBS, the cells were then stained by MF3Ec‐TBPP NPs (10 µg·mL^−1^) for 20 min. The washed cells were irradiated under white light (350−800 nm, 20 mW·cm^−2^) or placed in the dark environment for 30 min and imaged using a confocal microscope. For DCFH‐DA, the cell images were captured under 488 nm excitation with 2% laser power and the emission at 500−550 nm was collected. The emission intensity of DCF in the test groups was compared to the control group to assess ROS generation.

### The Cell Viability Test (MTT Essay)

MCF‐7 or SK‐BR‐3 cells were seeded at a density of 2 × 10^4^ cells·mL^−1^ in DMEM supplemented with 10% FBS and 1% penicillin‐streptomycin. The cells were cultured in a 96‐well plate at 37 °C in a 5% CO_2_ humidified incubator for 24 h. After incubation, the medium was replaced with 100 µL of fresh DMEM containing MF3Ec‐TBPP NPs at concentrations of 1, 2, 5, 10, 15, and 20 µg·mL^−1^. Following an additional 24 h of incubation, 10 µL of MTT solution (5 mg·mL^−1^ in PBS) was added to each well. After 4 h, 100 µL of DMSO was added to dissolve the formed formazan crystals, and the absorbance was measured at 595 nm using a plate reader.

### Evaluation of Photobleaching In Vitro

MCF‐7 cells were incubated with either MF3Ec‐Alexa488 (2 × 10^−2^ м) or MF3Ec‐TBPP NPs (10 µg·mL^−1^) at 37 °C for 30 min in DMEM supplemented with 10% FBS and 1% PS. Following incubation, the cells were washed twice with PBS to remove any excess dye. The washed cells were then irradiated with a 488 nm laser at 2% power, and fluorescence imaging was performed using a confocal microscope. Images were captured at 5‐min intervals over a 30‐min period. For MF3Ec‐Alexa488, the excitation wavelength was set to 488 nm with 2% laser power, and emission was collected in the range of 500–525 nm. For MF3Ec‐TBPP NPs, emission was collected in the range of 605−700 nm. The emission intensity of both MF3Ec‐Alexa488 and MF3Ec‐TBPP NPs was analyzed with varying exposure times to laser irradiation.

### Tumor‐Bearing Mouse Model and MF3Ec‐TBPP NPs Treatment

For the tumor‐cell‐bearing mouse model, 5 × 10^6^ MCF‐7 cells per site were implanted subcutaneously into the axillary breast fat pads of female BALB/cAnU‐nu nude mice. The breast cancer cell‐bearing mice were randomly divided into different groups, when the tumors reached 100 mm^3^, the mice were intravenously injected with 100 µL PBS or MF3Ec‐TBPP NPs (0.5 mg·mL^−1^), with white light illumination (100 mW·cm^−2^, 30 min) or under dark conditions once every 2 days for 12 days. Tumor size and body weight were monitored twice a week, and tumor volume was calculated using the formula (width × width × length) × 0.5. Experiments involving animals were approved by the licensing Committee on the Use of Live Animals in Teaching and Research (CULATR No. 4484‐17 and 5420‐20) of the University of Hong Kong.

### Hematoxylin and Eosin (H&E) Staining

At day 12 after NPs treatment, the mice were sacrificed, and tumor tissues were photographed. The tumor tissues and organs including heart, liver, spleen, lung, and kidney were collected, fixed in 4% paraformaldehyde (PFA), and embedded in paraffin. The 4–5 µm sections were made by microtome (Leica, Germany) and adhered to on slides for H&E staining procedures including dewaxing, hydration, hematoxylin staining, washing, eosin staining, dehydration, and sealing. Images were captured by optical microscopy (Olympus BX43, Japan).

### In Vivo Fluorescent Imaging and Organ Distribution Study

To evaluate the in vivo targeting effects, two different types of breast cancer cells (5 × 10^6^ MCF‐7 and 10^7^ SKBR3 cells per site) were respectively implanted subcutaneously into the right and left sides of axillary breast fat pads of each female BALB/cAnU‐nu nude mouse. Ex vivo fluorescent imaging of tumor‐bearing nude mice at 24 h after tail intravenous injection of different volumes (40, 80, and 120 µL) MF3Ec‐TBPP NPs (0.5 mg·mL^−1^) were obtained on a small animal imaging system (PE IVIS Spectrum). The excitation wavelength was 500 nm, and the fluorescence was collected at 700 nm. After intravenous injection of 100 µL of MF3Ec‐TBPP NPs (0.5 mg·mL^−1^), in vivo fluorescent images of the tumor‐bearing mouse were captured at 0.5, 24, and 48 h, with the fluorescence background from the NPs subtracted on the same imaging system. After 48 h of injection, the mice were sacrificed and tumors and organs (heart, liver, spleen, lung, kidney) were collected to observe the organ distribution. The samples were also assessed on the same imaging system.

### Hemolysis Assay

Fresh mice blood was stabilized with EDTA coagulation tube to prevent coagulation. RBCs were isolated by centrifugation at 1500 g for 10 min at 4 °C, and the resulting pellet was washed five times with 1× PBS to remove remaining plasma and other soluble components. After washing, the RBC pellet was diluted with PBS at a ratio of 1:10 to prepare the RBC suspension. For the hemolysis assay, 0.1 mL of the RBC suspension was mixed with either 0.9 mL 1× PBS (negative control), 0.9 mL distilled water (positive control), or 0.9 mL dispersions of MF3Ec‐TBPP NPs at concentrations ranging from 10 to 100 µg·mL^−1^ in PBS. The mixtures were incubated at 37 °C with shaking at 200 rpm for 1 h to promote efficient interaction between the NPs and RBCs. After incubation, the mixtures were centrifuged at 2500 g for 5 min, and the supernatants were collected to measure the absorbance of released hemoglobin at 576 nm using a plate reader. The hemolysis rate was calculated using Equation (2):

(2) $$\begin{array}{cc} & \mathrm{Hemolysis}\;\mathrm{rate}\;\left(\%\right)\\ & \;=\frac{\left[\left(\mathrm{sample}\;\mathrm{absorbance}-\mathrm{negative}\;\mathrm{control}\;\mathrm{absorbance}\;\right)\right.}{\left.\left(\mathrm{positive}\;\mathrm{control}\;\mathrm{absorbance}-\mathrm{negative}\;\mathrm{control}\;\mathrm{absorbance}\right.\right]}\times 100 \end{array}$$

The sample absorbance values were corrected by subtracting the absorbance of the pure nanoparticle dispersions (measured in PBS for the negative control and in DI water for the positive control) to ensure accurate results and eliminate any interference caused by MF3Ec‐TBPP NPs.

### Statistical Analysis

All quantitative data were expressed as mean ± standard deviation (SD). Statistical comparisons were performed using one‐way ANOVA in GraphPad Prism 8. Statistical significance was indicated as follows: *p < 0.05, **p < 0.01, ***p < 0.001.

## Conflict of Interest

The authors declare no conflict of interest.

## Supporting information



Supporting Information

Supplemental Video 1

## Data Availability

The data that support the findings of this study are available in the supplementary material of this article.
