# Supplementary material for: Strategic Design of Aptamer‐Guided Aggregation‐Induced Emission Nanoparticles for Targeted Photodynamic Therapy in Breast Cancer
Source: Adv Sci (Weinh). 2025 Sep 9;12(43):e03358. doi: 10.1002/advs.202503358 (PMC12631910; doi:10.1002/advs.202503358)
Supplement: Supplementary file 1 — Supporting Information [file ADVS-12-e03358-s002.docx]

Supporting Information

**Strategic Design of Aptamer-Guided Aggregation-Induced Emission Nanoparticles for Targeted Photodynamic Therapy in Breast Cancer**

*Charlie C. H. Ma, Qingqing Liu, Yue Sui, Wutong Du, Kristy W. K. Lam, Jacky W. Y. Lam, Chao Li, Tengteng Chen, Jianwei Sun, Nuomin Li, Ryan T. K. Kwok, Jianping Chen,* Feiyi Sun,* and Ben Zhong Tang**

**Supplementary Figures**

**Figure S1.** Synthetic route to TBPP.


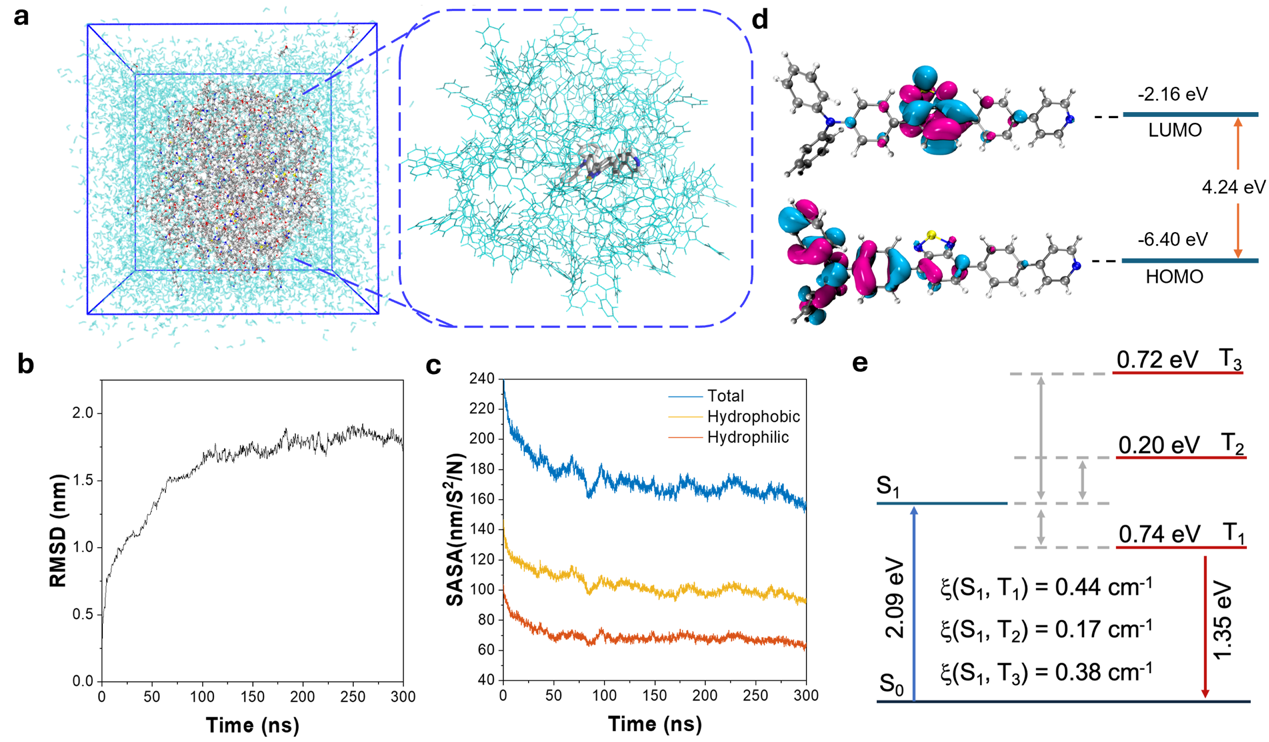


**Figure S2.** Theoretical calculations of TBPP aggregate formation and properties water/THF mixture (*f*_W_ = 80%). (a) Molecular dynamics simulation snapshots of TBPP aggregate formed in a water/THF mixture with 80% *f*_W_, the innermost TBPP molecule was shown using the CPK model. (b) Root mean square deviation (RMSD) and (c) solvent-accessible surface area (SASA) for the TBPP aggregates. (d) Frontier molecular orbitals and corresponding energy levels, and calculated *ΔE* in ground state. (e) Energy levels with SOC in excited state of the innermost TBPP molecule in the ground state in aggregate at M062X/6-311G** level.


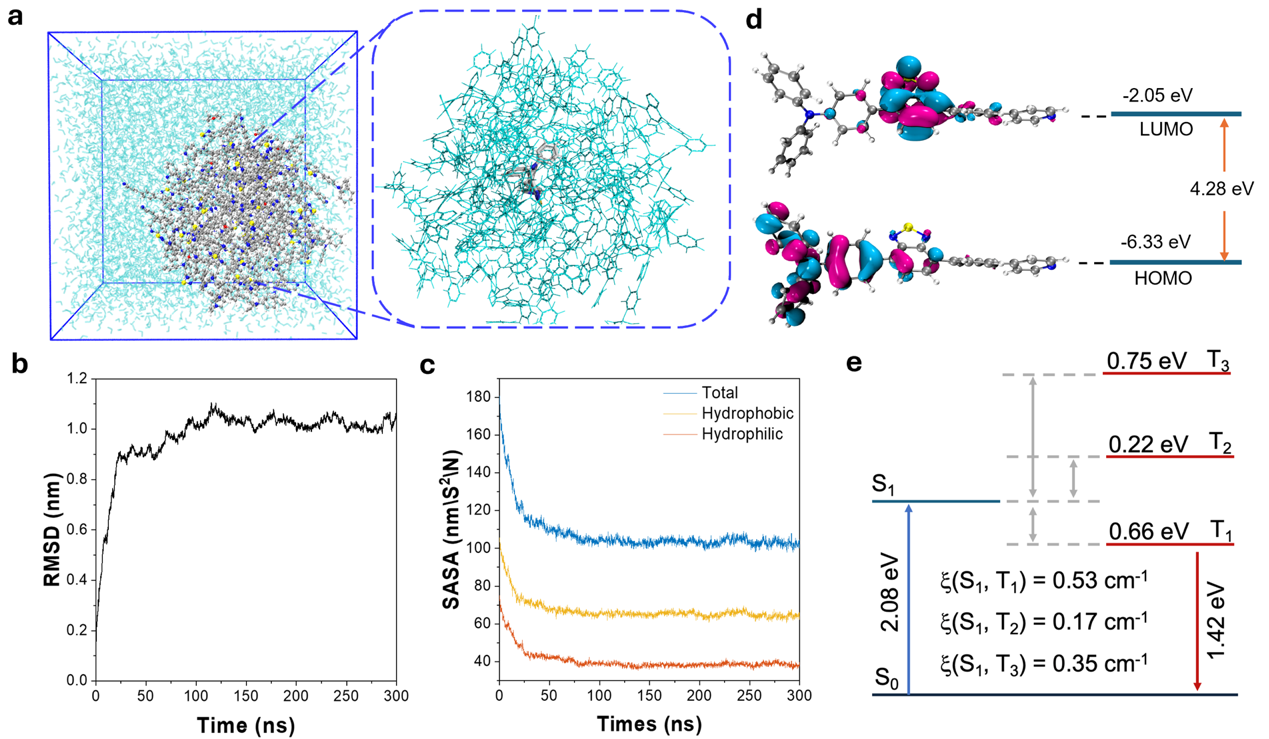


**Figure S3.** Theoretical calculation of TBPP on aggregate formation and properties in a water/THF mixture (*f*_W_ = 99%). (a) Molecular dynamics simulation snapshots of TBPP aggregate formed in a water/THF mixture with 99% *f*_W_. The innermost TBPP molecule was shown using the CPK model. (b) RMSD and (c) SASA for the TBPP aggregate.
(d) Frontier molecular orbitals and corresponding energy levels, and calculated *ΔE* in ground state. (e) Energy levels with SOC in excited state of the innermost TBPP molecule in the ground state in aggregate at M062X/ 6-311G** level.

**
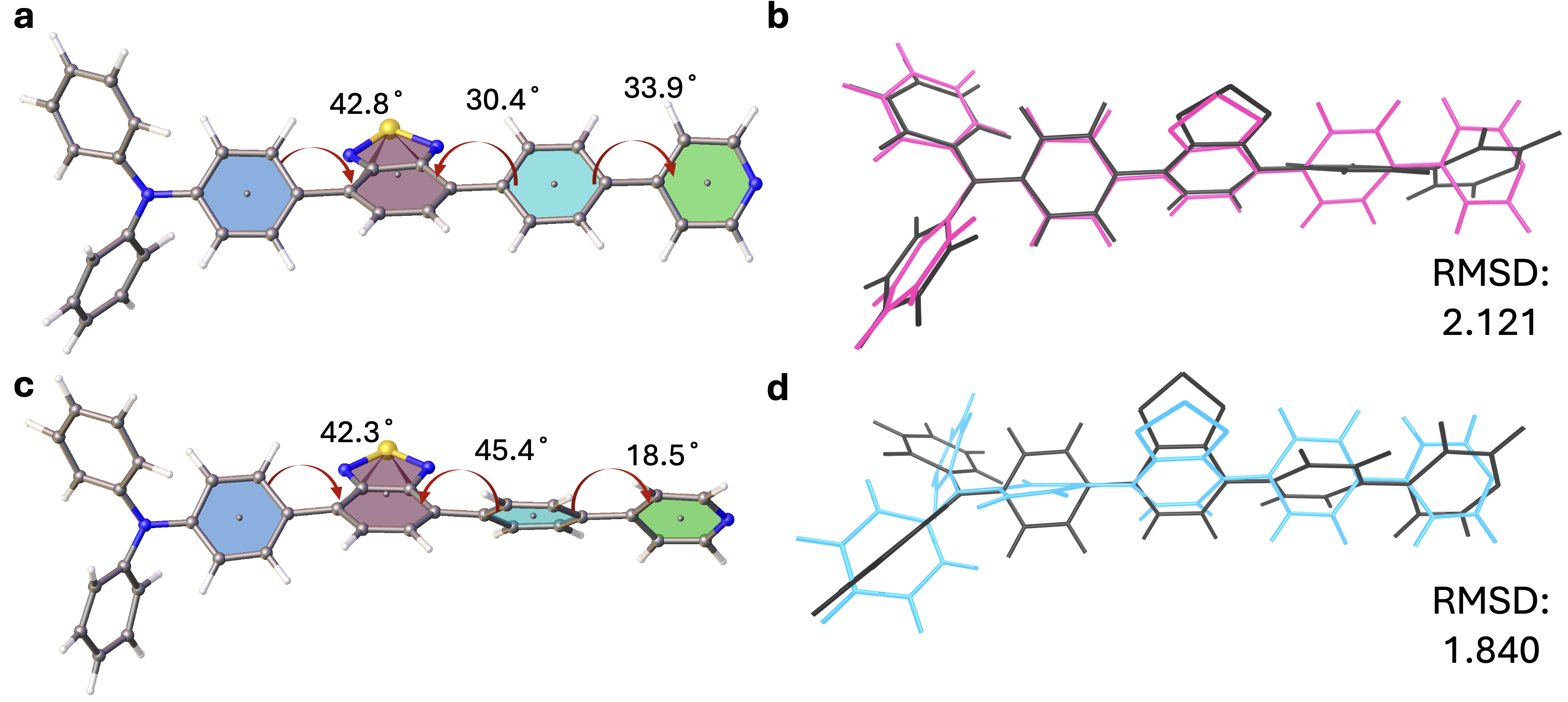
**

**Figure S4.** Molecular spatial configuration, dihedral angles and comparison with the optimized configuration of the single molecule ground state in 80% (a, b) and 99% (c, d) H_2_O/THF mixture at M062X/6-311G** level.


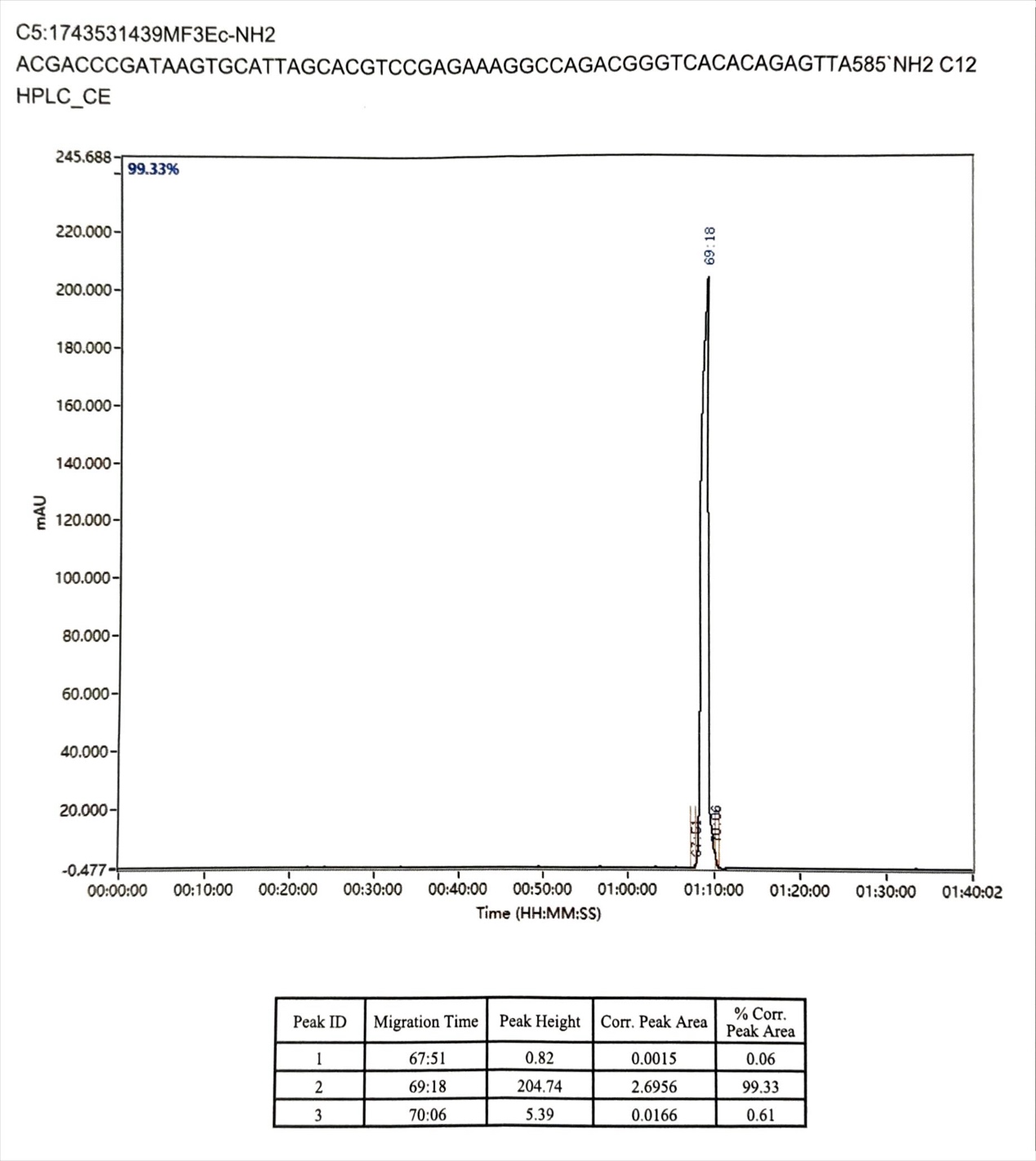


**Figure S5**. Purification and characterization of the MF3Ec ssDNA aptamer. The HPLC analysis of the amine-conjugated MF3Ec ssDNA aptamer (MF3Ec-NH_2_) provided by ATCG Limited. Purification and characterization of oligonucleotide were performed by WAVE system.

**
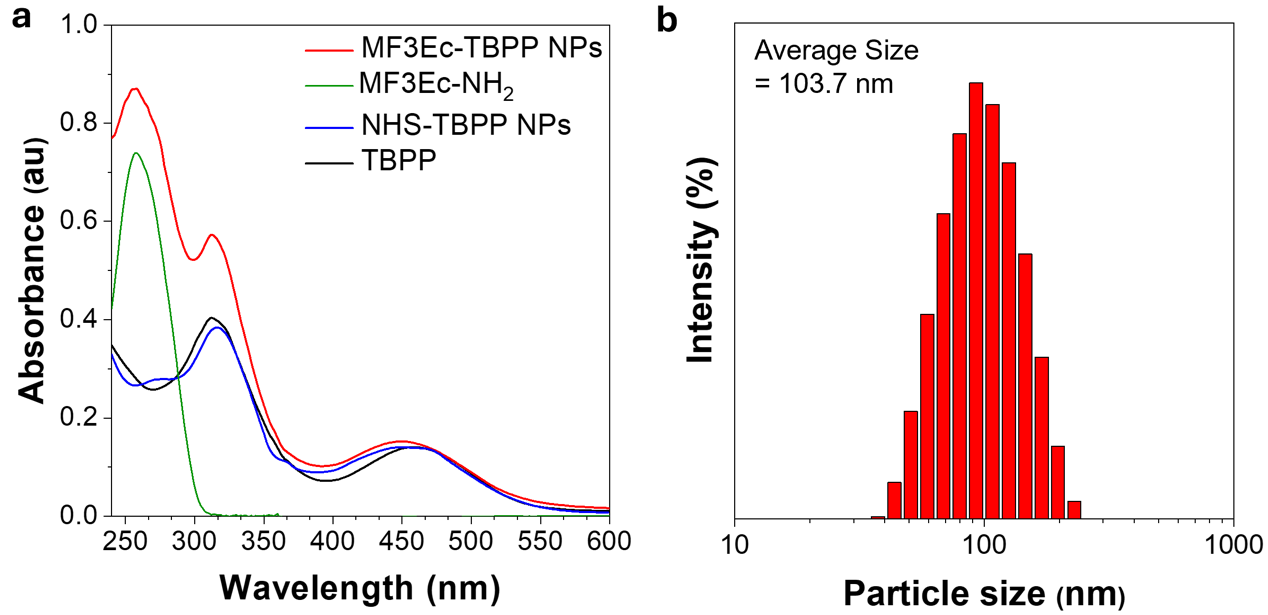
**

**Figure S6.** Characterization of TBPP NPs. (a) UV-Vis absorption spectra of MF3Ec aptamer, TBPP aggregates and TBPP NPs. [TBPP] = [MF3Ec-TBPP NPs] = [NHS-TBPP NPs] = 1 ×10^-5^ м; [MF3Ec-NH_2_] = 1 × 10^-6^ м. (b) DLS measurement of NHS-TBPP NPs.


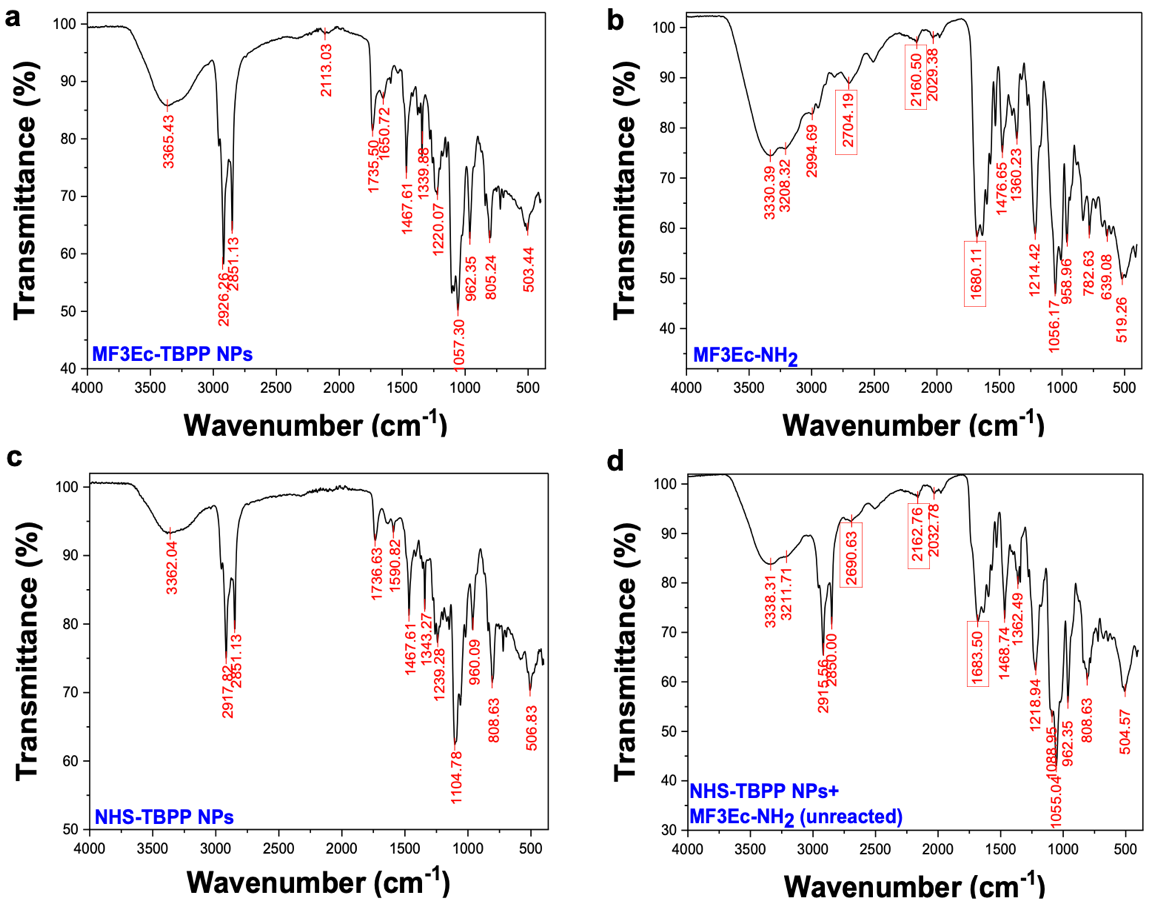


**Figure S7.** FTIR spectra of mixtures of (a) MF3Ec-TBPP NPs, (b) MF3Ec-NH_2_, (c) NHS-TBPP NPs and (d) the unreacted mixture of NHS-TBPP NPs and MF3Ec-NH_2_.


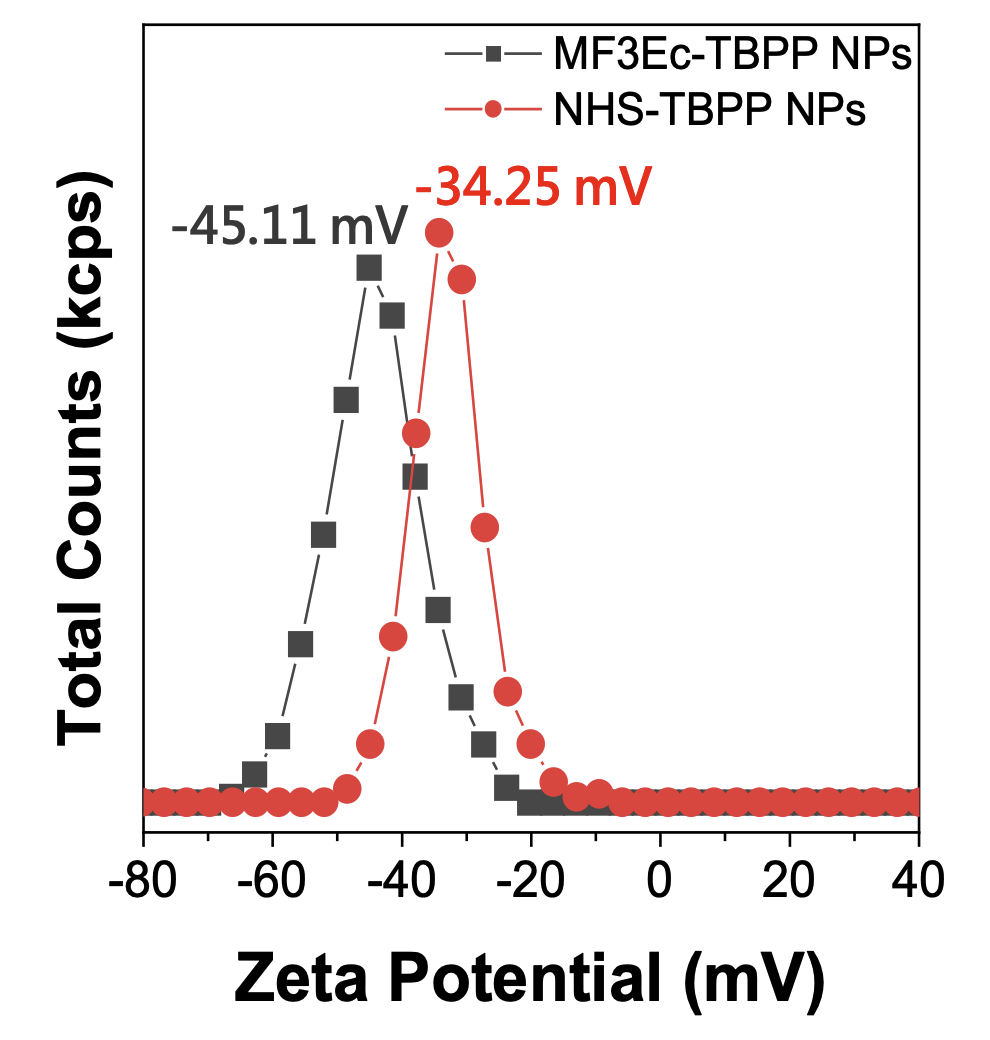


**Figure S8.** Zeta potential measurement of NHS-TBPP NPs and MF3Ec-TBPP NPs.


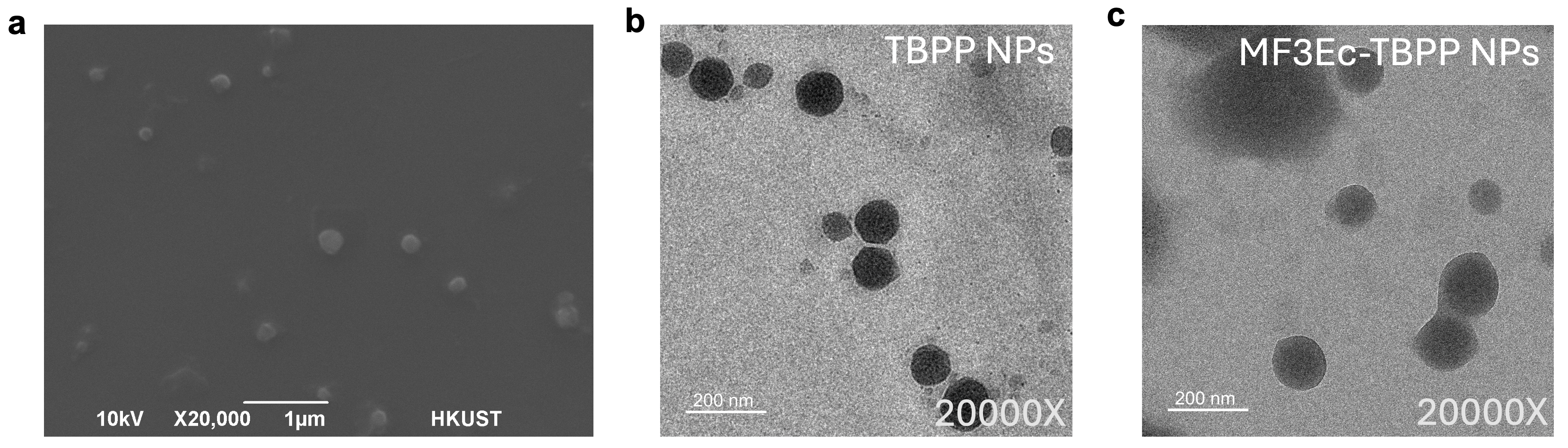


**Figure S9.** (a) SEM image of TBPP NPs at 20000× magnification. Scale bar = 1 μm. (b and C) TEM images of (b) TBPP NPs and (c) MF3Ec-TBPP NPs at 20000× magnification. Scale bar = 200 nm.


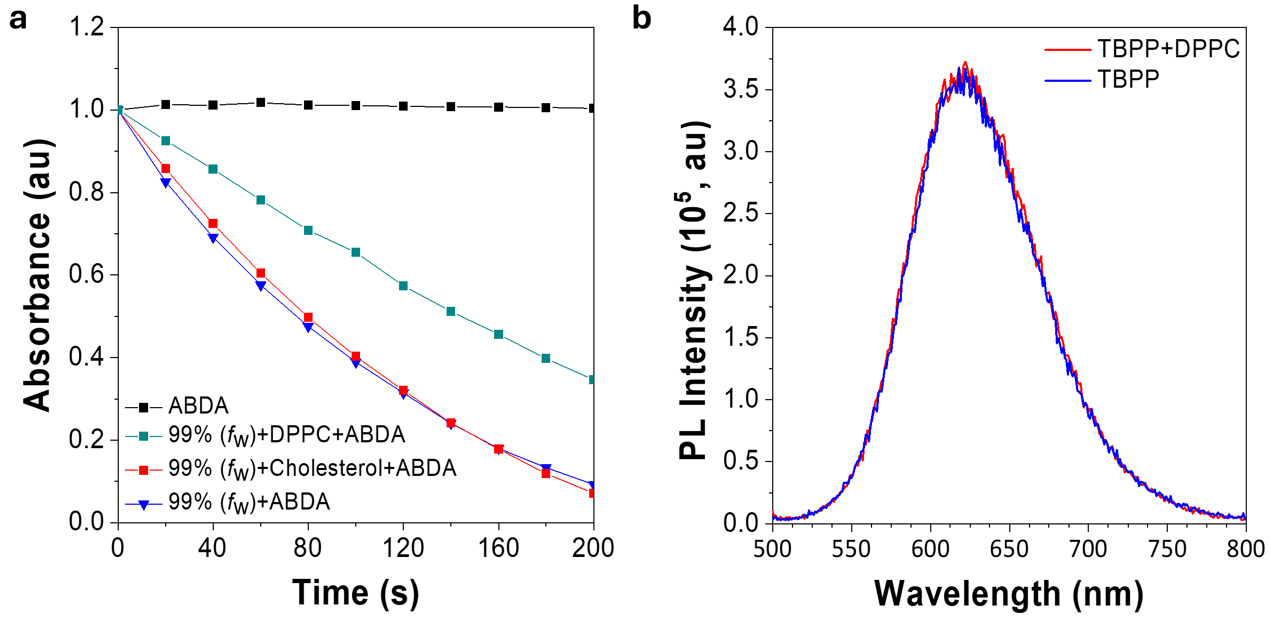


**Figure S10.** Photophysical behavior of DPPC and cholesterol mixed with TBPP aggregates (a) The rate of absorbance reduction of ABDA at 378 nm measured in the mixutres of TBPP aggregates (*f*_W_ = 99%) and ABDA containing DPPC or cholesterol under white light irradiation (10 mW·cm^-2^). [ABDA] = [DPPC] = 1 × 10^-4^ м, [Cholesterol] = 50 mM. (b) PL intensities of TBPP aggregates in the presence or absence of DPPC. [TBPP] = 1 ×10^-5^ м, [DPPC] = 1 ×10^-4^ м.


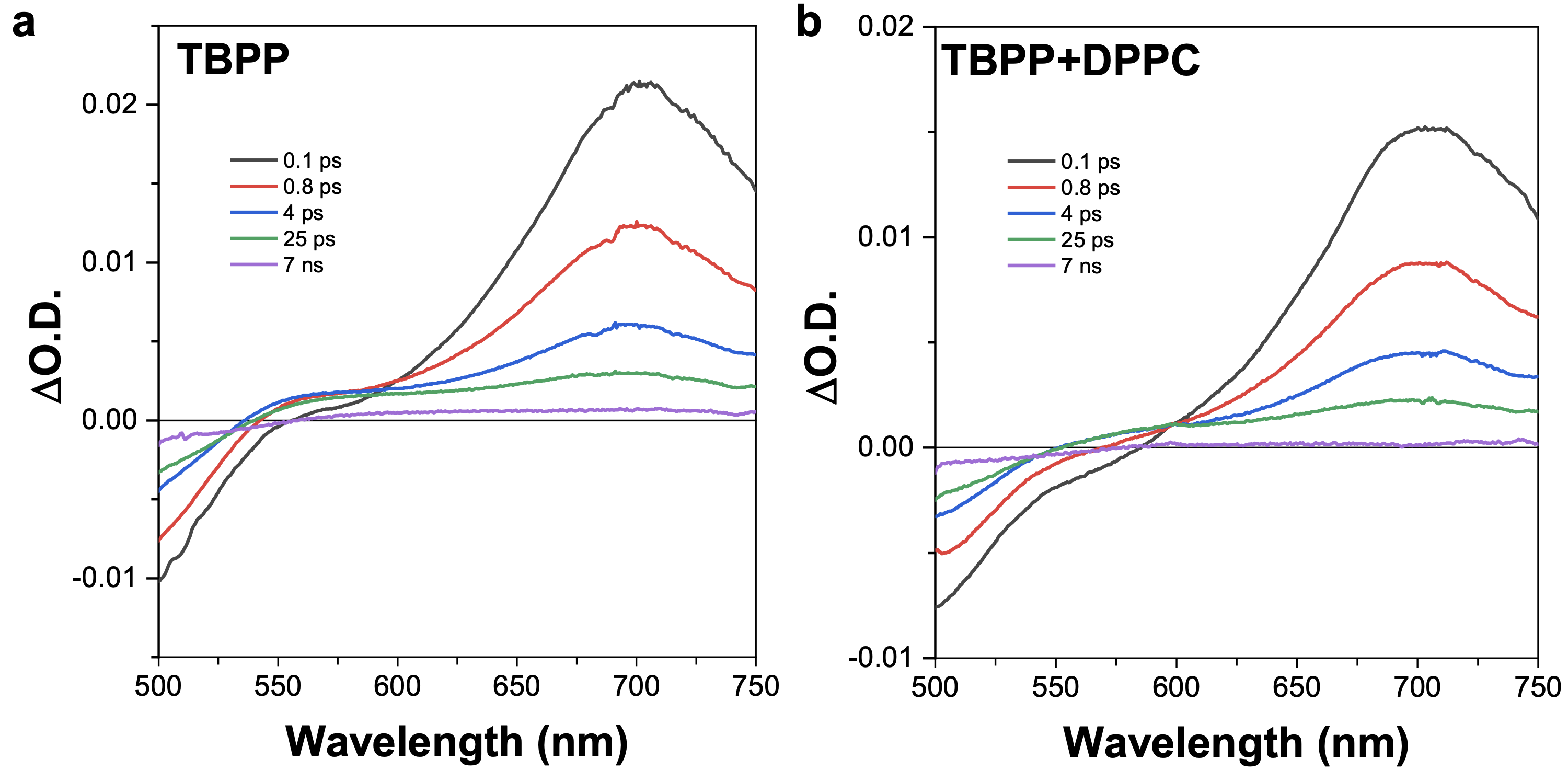


**Figure S11.** The fs-TA spectra of (a) TBPP aggregates (*f_W_* = 99%) and (b) TBPP aggregates (*f*_W_ = 99%) in the presence of DPPC (100 µM) at selected pump-probe time delays upon excitation at 470 nm with a power of 100 μW.


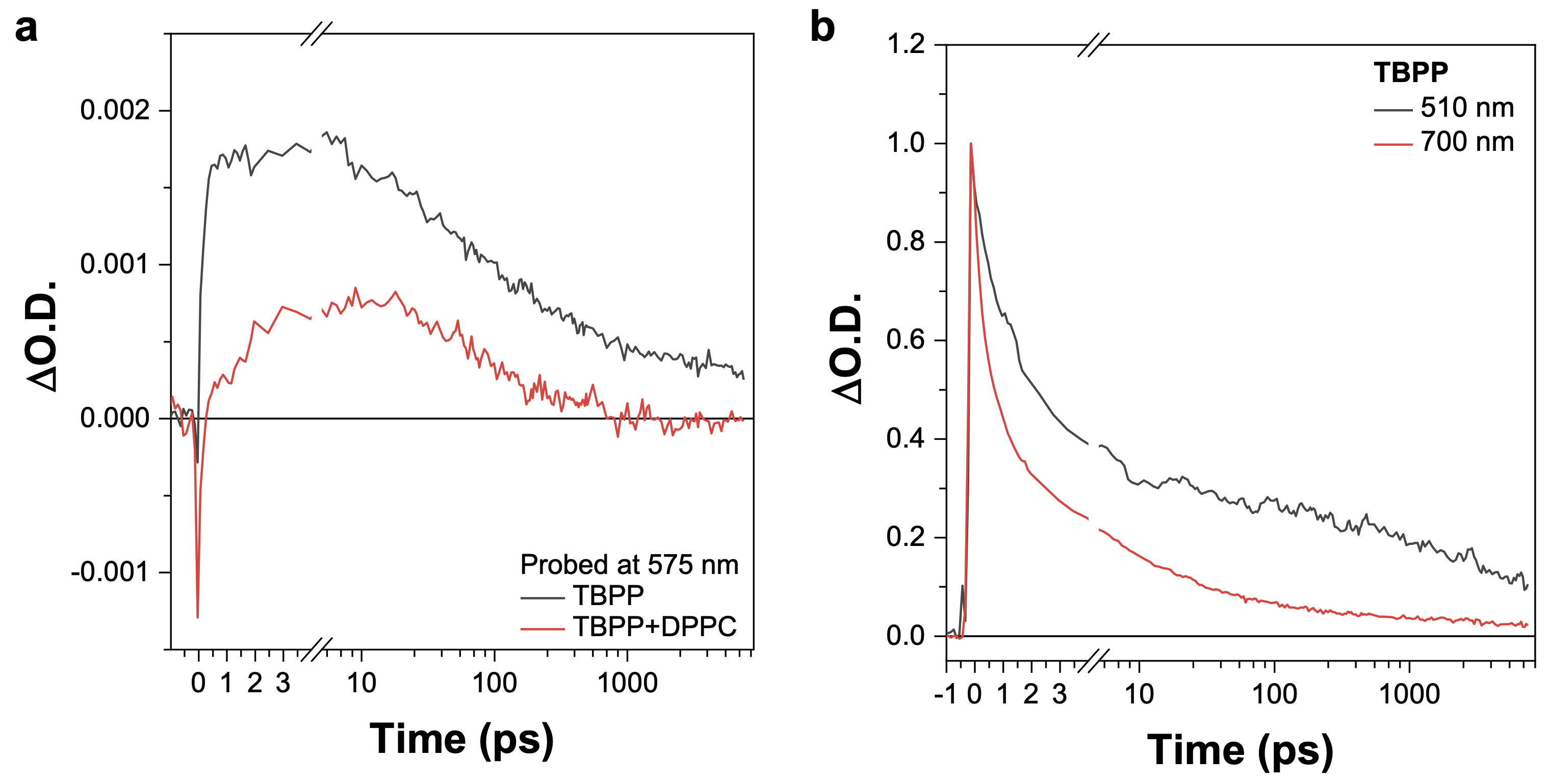


**Figure S12. (**a) The normalised single kinetic traces of TBPP aggregates (*f*_W_ = 99%) probed at GSB (510 nm) and singlet excited state absorption (700 nm). (b) The single kinetic traces of TBPP aggregates (*f*_W_ = 99%) in the absence and presence of DPCC (100 µM) probed at triplet excited state absorption (575 nm).


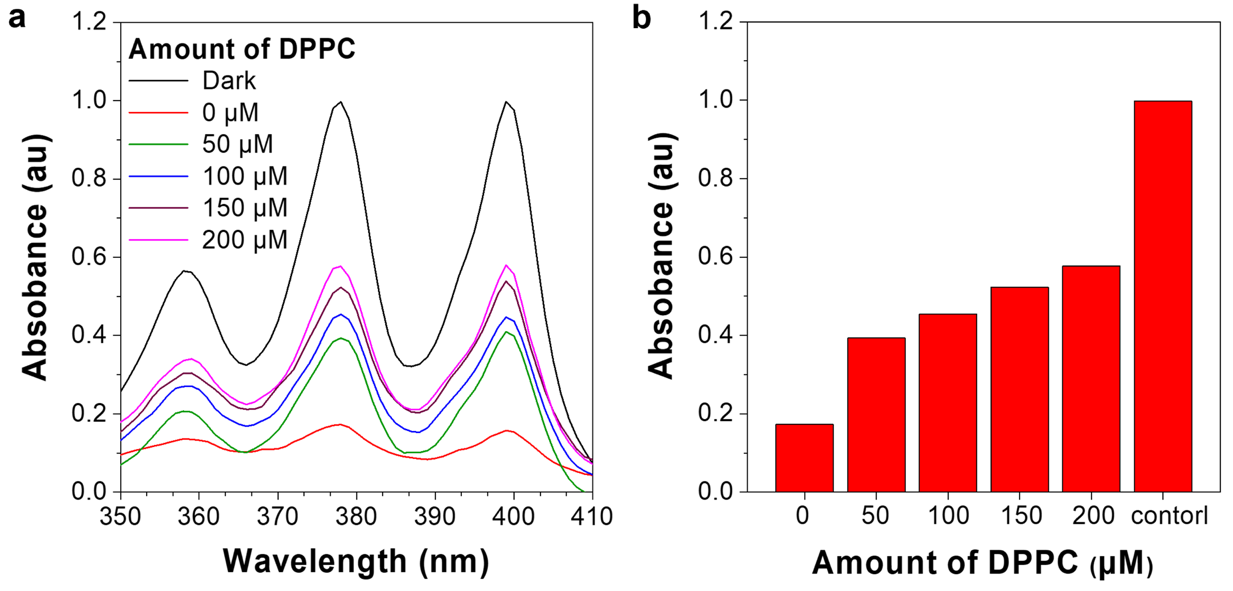


**Figure S13.** Effects of DPPC on ^1^O_2_ generation by TBPP aggregates (*f*_W_ = 99%). (a) The UV-Vis spectra of TBPP aggregates containing ABDA and varying concentrations of DPPC. (b) The change in absorbance at 378 nm of the mixtures of TBPP aggregates (*f*_W_ = 99%), ABDA and different amount of DPPC. [ABDA] = 1 × 10^-4^ м, [TBPP] = 1 × 10^-5^ м. These measurements were performed under white light irradiation (10 mW·cm^-2^).


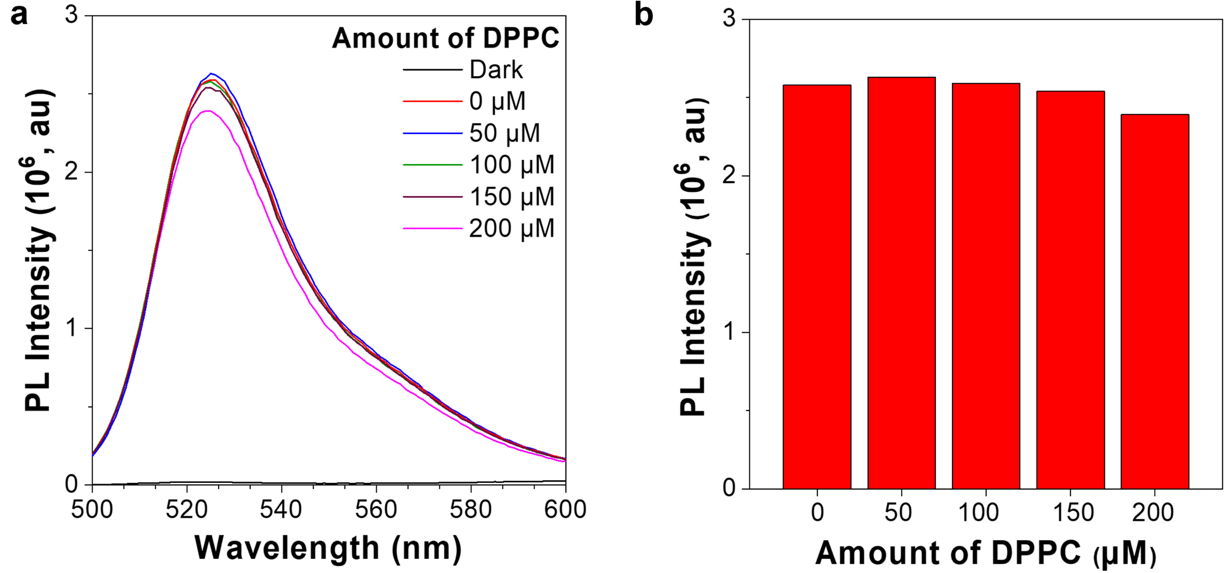


**Figure S14.** Effects of DPPC on total ROS generation by TBPP aggregates (*f*_W_ = 99%). (a) PL spectra of TBPP aggregates containing DCFH and varying concentration of DPPC under white light irradition (10 mW·cm^-2^) for 200 s. (b) The change in PL intensity at 535 nm of the mixtures of TBPP aggregates, DCFH and different amount of DPPC under white light irradition. [DCFH] = 5 × 10^-5^ м, [TBPP] = 1 × 10^-5^ м, *λ*_ex_ = 490 nm.


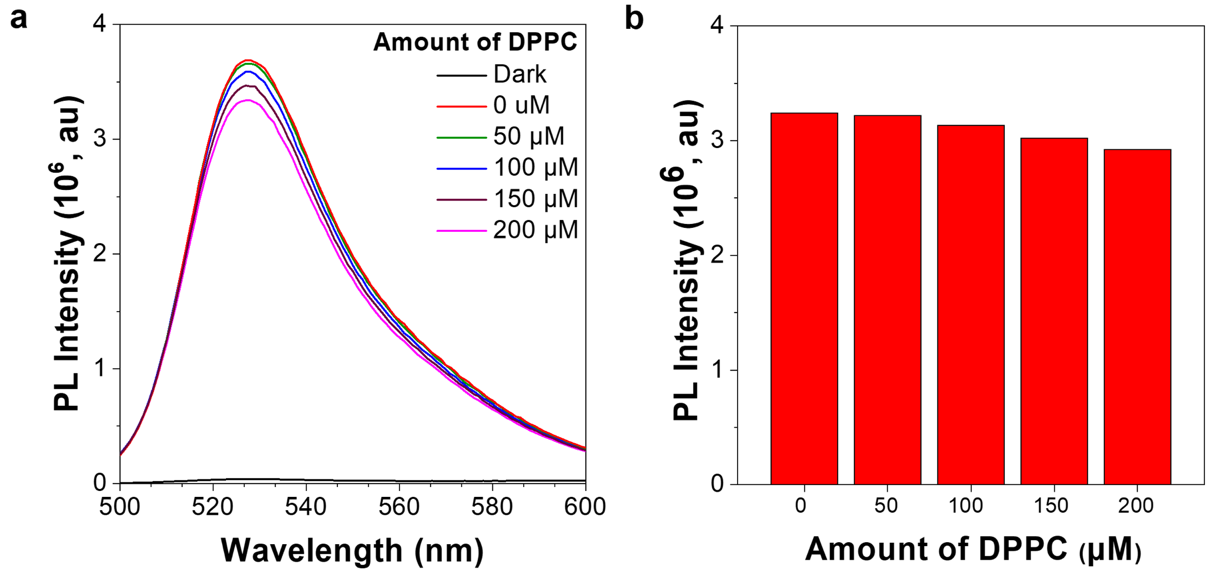


**Figure S15.** Effects of DPPC on O_2_^•-^ generation by TBPP aggregates (*f*_W_ = 99%). (a) PL spectra of TBPP aggregates containing DHR123 and different amounts of DPPC under white light irradiation (10 mW·cm^-2^) for 200 s. (b) The change in PL intensity at 525 nm of the mixtures containing TBPP aggregates, DHR123 and different amounts of DPPC. [DHR123] = [TBPP] = 1 × 10^-5^ м, *λ*_ex_ = 488 nm.


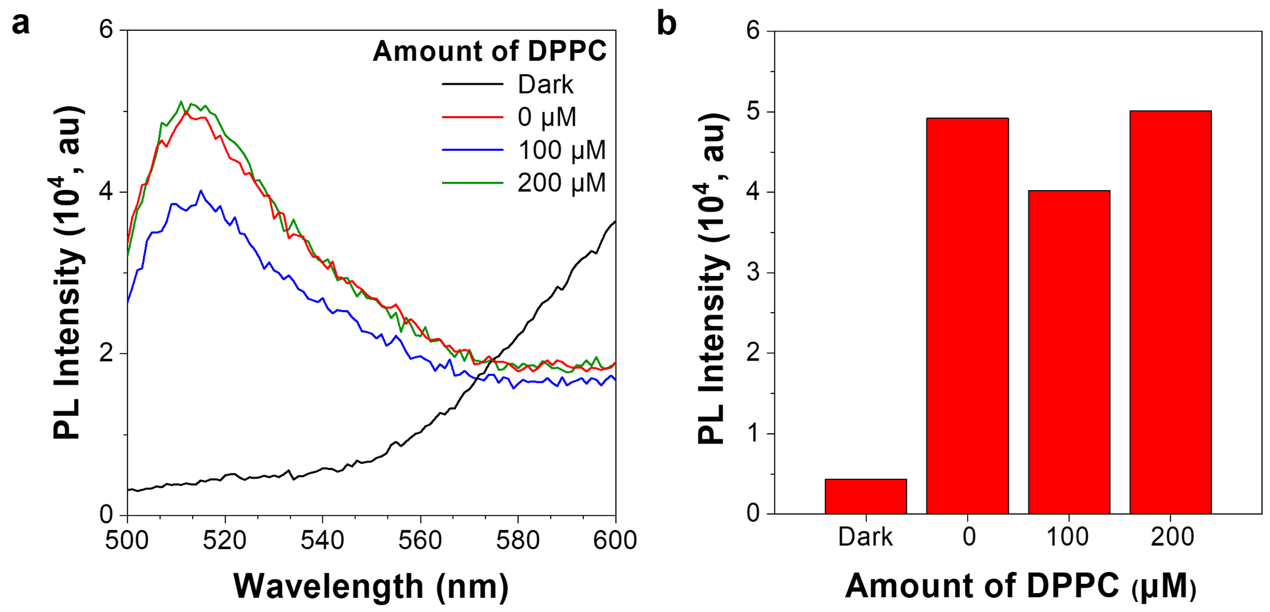


**Figure S16.** Effects of DPPC on OH• generation by TBPP aggregates (*f*_W_ = 99%). (a) PL spectra of TBPP aggregates containing HPF and different amounts of DPPC under white light irradition (10 mW·cm^-2^) for 200 s. (b) The change in PL intensity at 515 nm of the mixtures containing TBPP aggregates, HPF and different amounts of DPPC. [HPF] = [TBPP] = 1 × 10^-5^ м, *λ*_ex_ = 490 nm.


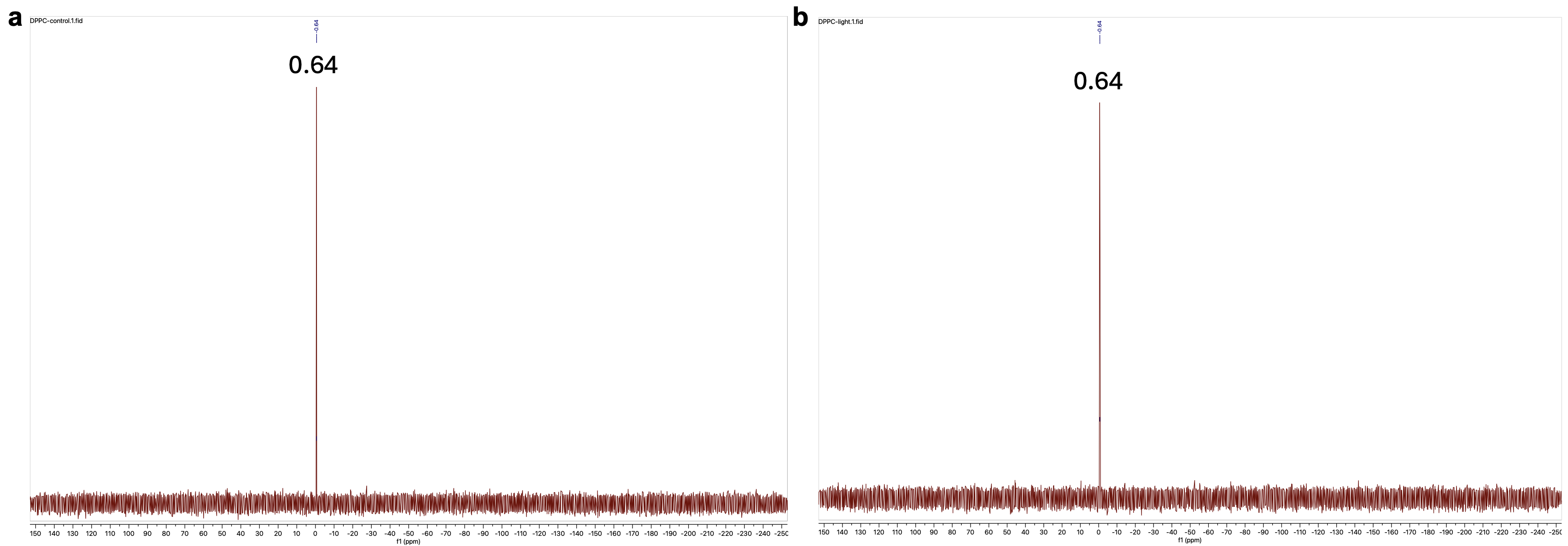


**Figure S17.** Evaluation of structural change after prolonged irradiation. ^31^P NMR spectrum of the mixture of DPPC and TBPP aggregates (*f*_W_ = 99%) recorded (a) before and (b) after white light irradiation (20 mW·cm^-2^) for 1 h.

**
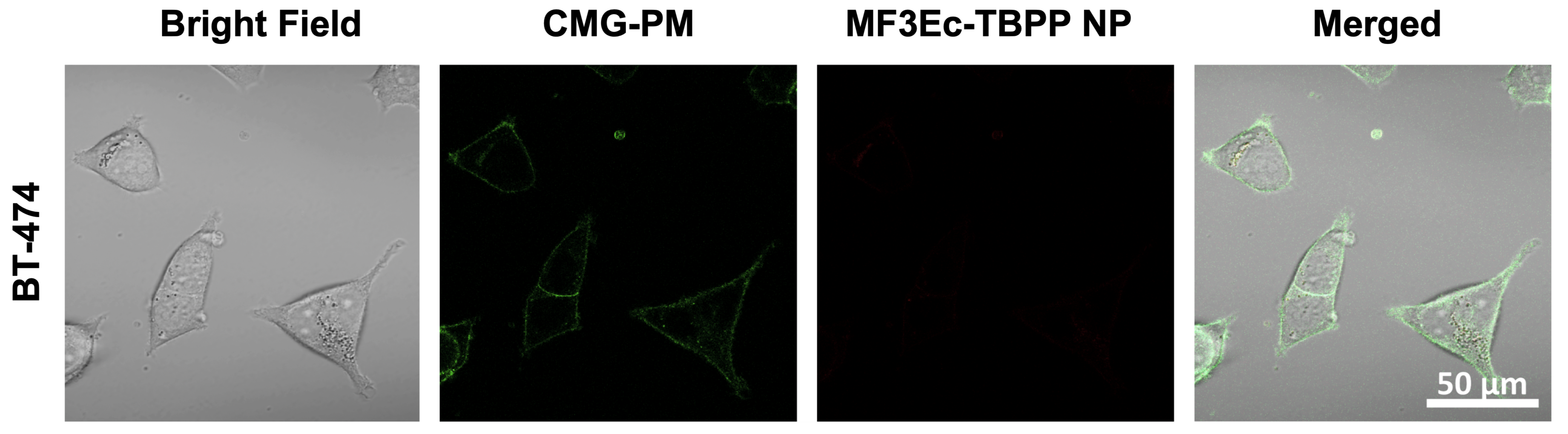
**

**Figure S18.** In vitro imaging of MF3Ec-TBPP NPs in luminal B type BC. (a) Bright-field and CLSM images of BT-474 cells incubated with MF3Ec-TBPP NPs (10 μg·ml^-1^) and CellMask^TM^ green plasma membrane stain (CMG-PM, 1×) for 15 min.


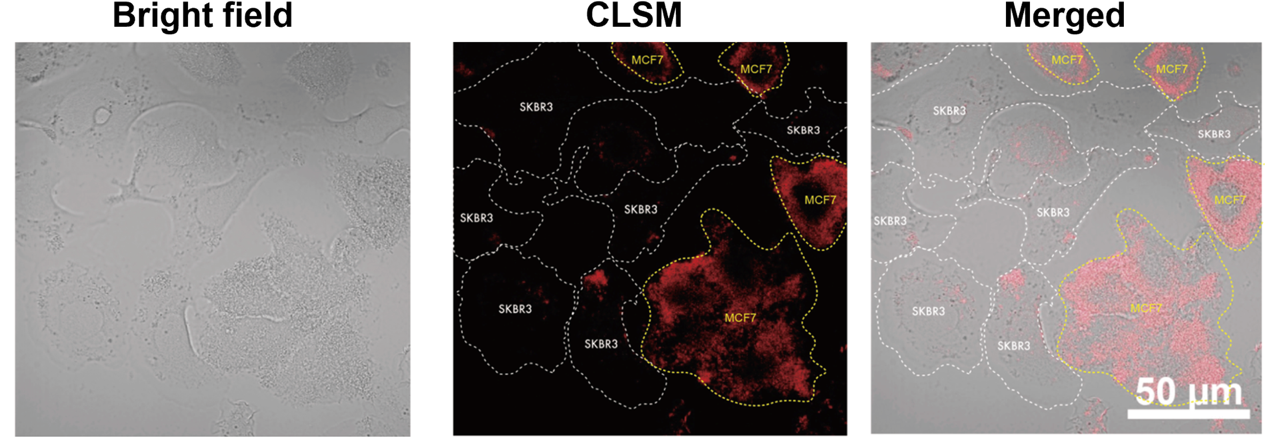


**Figure S19*.*** Bright-field and CLSM images of co-cultured MCF-7 and SK-BR-3 cells after staining with MF3Ec-TBPP NPs. The co-cultured cells were incubated 10 μg·ml^-1^ of MF3Ec-TBPP NPs for 15 mins. White dotted lines: SK-BR-3 cells; yellow dotted lines: MCF-7 cells. *λ*_ex_ = 488 nm; *λ*_em_ = 600−700 nm; objective lens = 40×; scale bar = 50 μm.


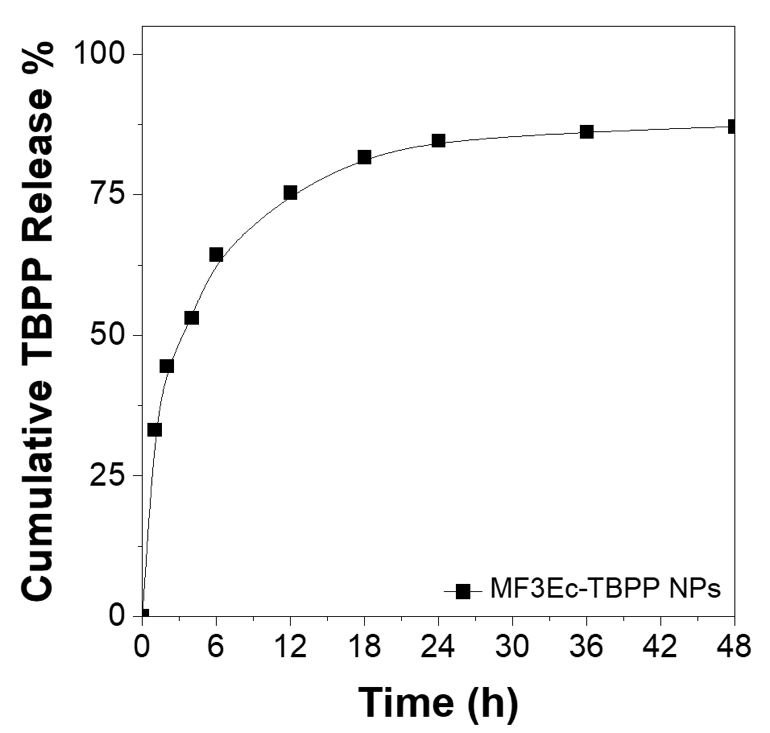


**Figure S20.** The release curves of TBPP from MF3Ec-TBPP NPs over time reflected by the change in absorbance of TBPP. NPs concentrations = 0.1 mg·ml^-1^.

**
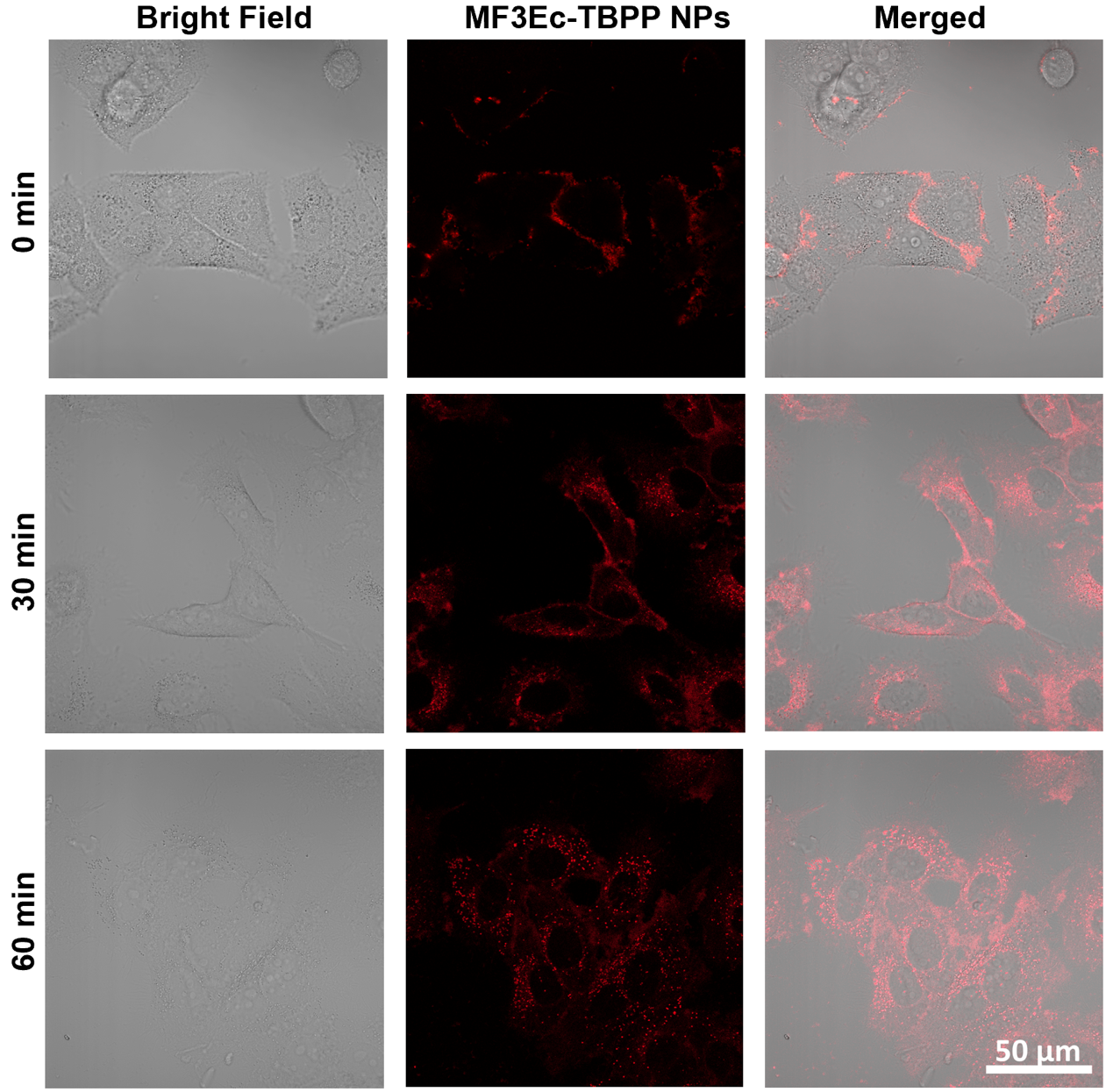
**

**Figure S21.** Time-series cell imaging of TBPP release and uptake from MF3Ec-TBPP NPs by MCF-7 cells. MCF-7 cells were incubated with 10 μg·ml^-1^ of MF3Ec-TBPP NPs for 20 min, followed by washing twice with 1 × PBS. The cells were then incubated in the dark for 0, 30, and 60 min, with imaging conducted at each time point. Objective lens = 40×; scale bar = 50 μm; *λ*_ex_ = 488 nm; MF3Ec-TBPP NPs: *λ*_em_ = 600−700 nm.

**
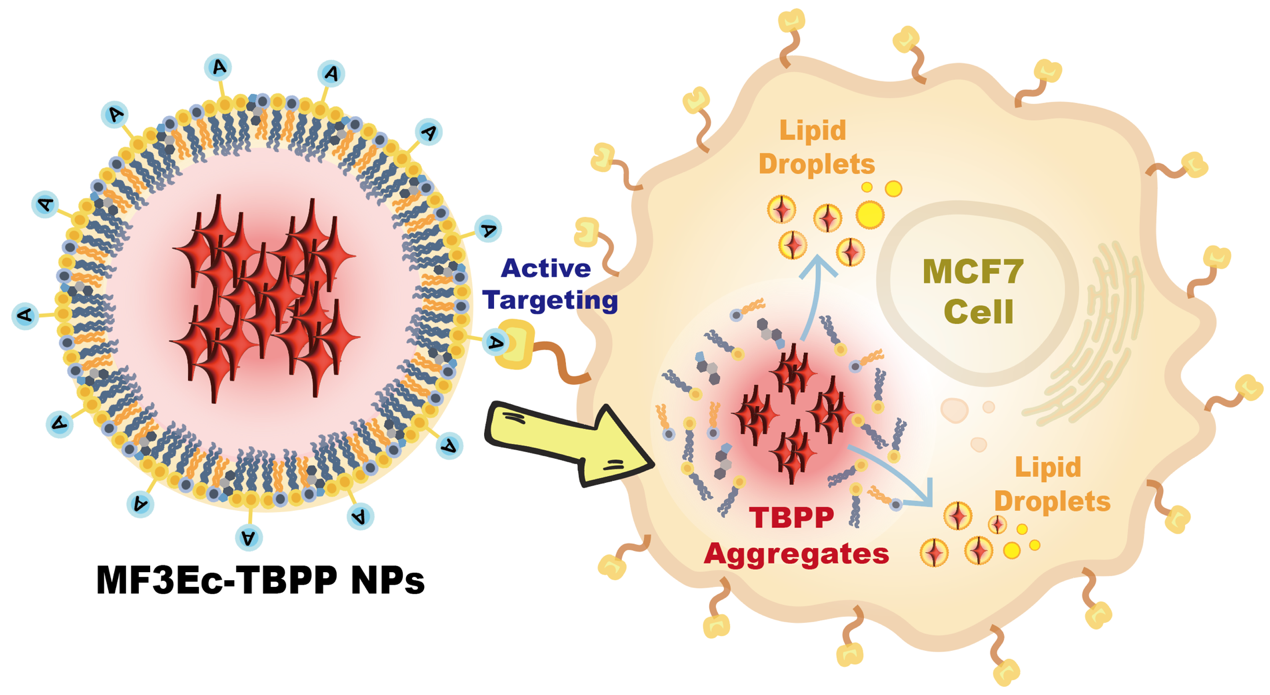
**

**Figure S22**. Schematic diagram illustrating active targeting and photodynamic therapy of MCF-7 breast cancer cell by MF3Ec-TBPP NPs.


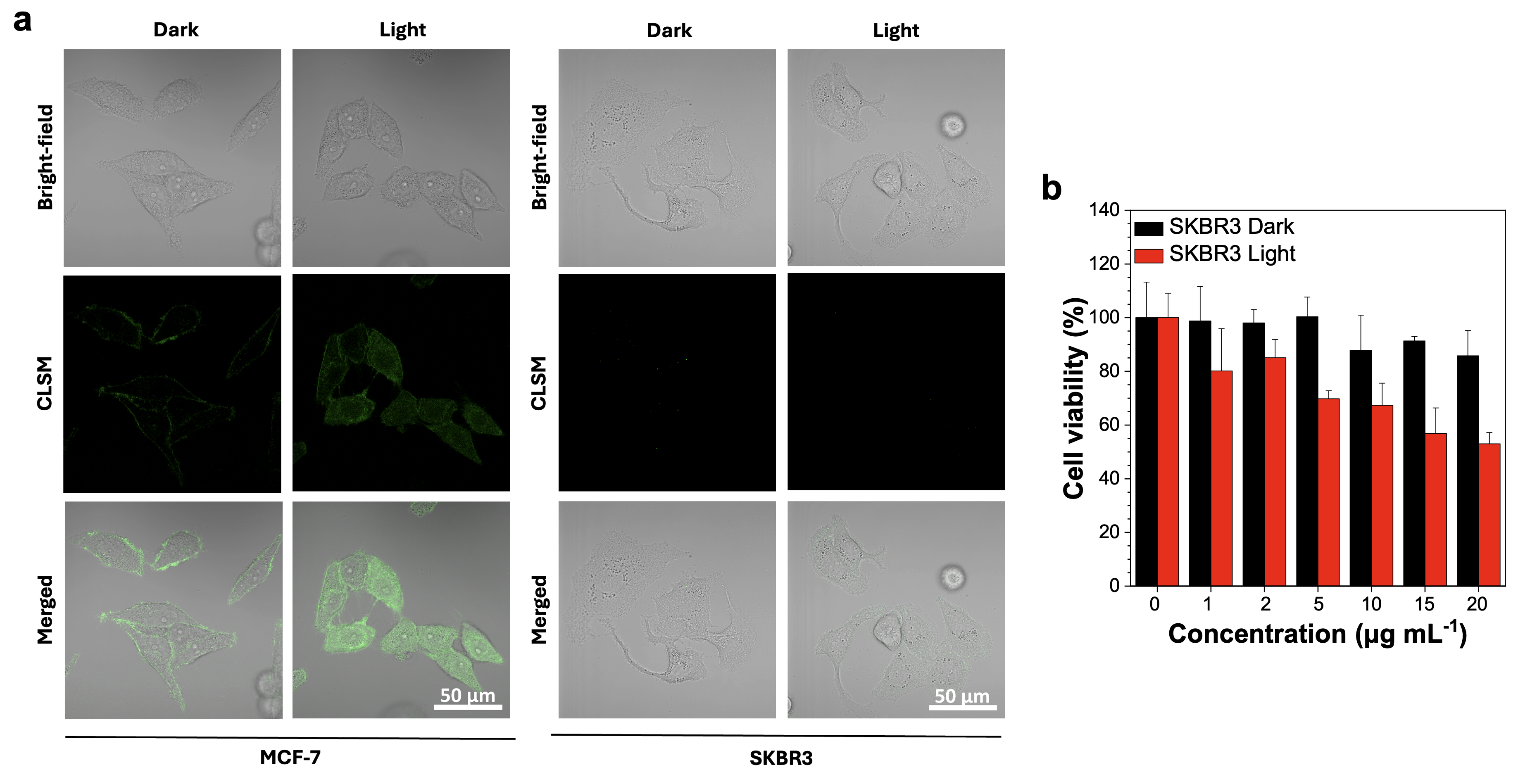


**Figure S23.** *In Vitro* imaging and PDT evaluation of MF3Ec-TBPP NPs. (a) Bright-field and CLSM images of MCF-7 or SK-BR-3 cells treated with DCFH-DA (0.1 mM) and MF3Ec-TBPP NPs (10 μg·ml^-1^) under white light irradiation (20 mW·cm^-2^) for 30 mins. λ_ex_ = 488 nm; λ_em_ = 500−550 nm; objective lens = 40×; scale bar = 50 μm. (b) MTT assay for SKBR3 cells incubated with MF3Ec-TBPP NPs in a dark environment or under white light irradiation (20 mW cm^-2^, 40 mins). All data shown are expressed as mean values ± standard deviation (Each group, n = 3). **p* < 0.5, ***p* < 0.01, and ****p* < 0.001.


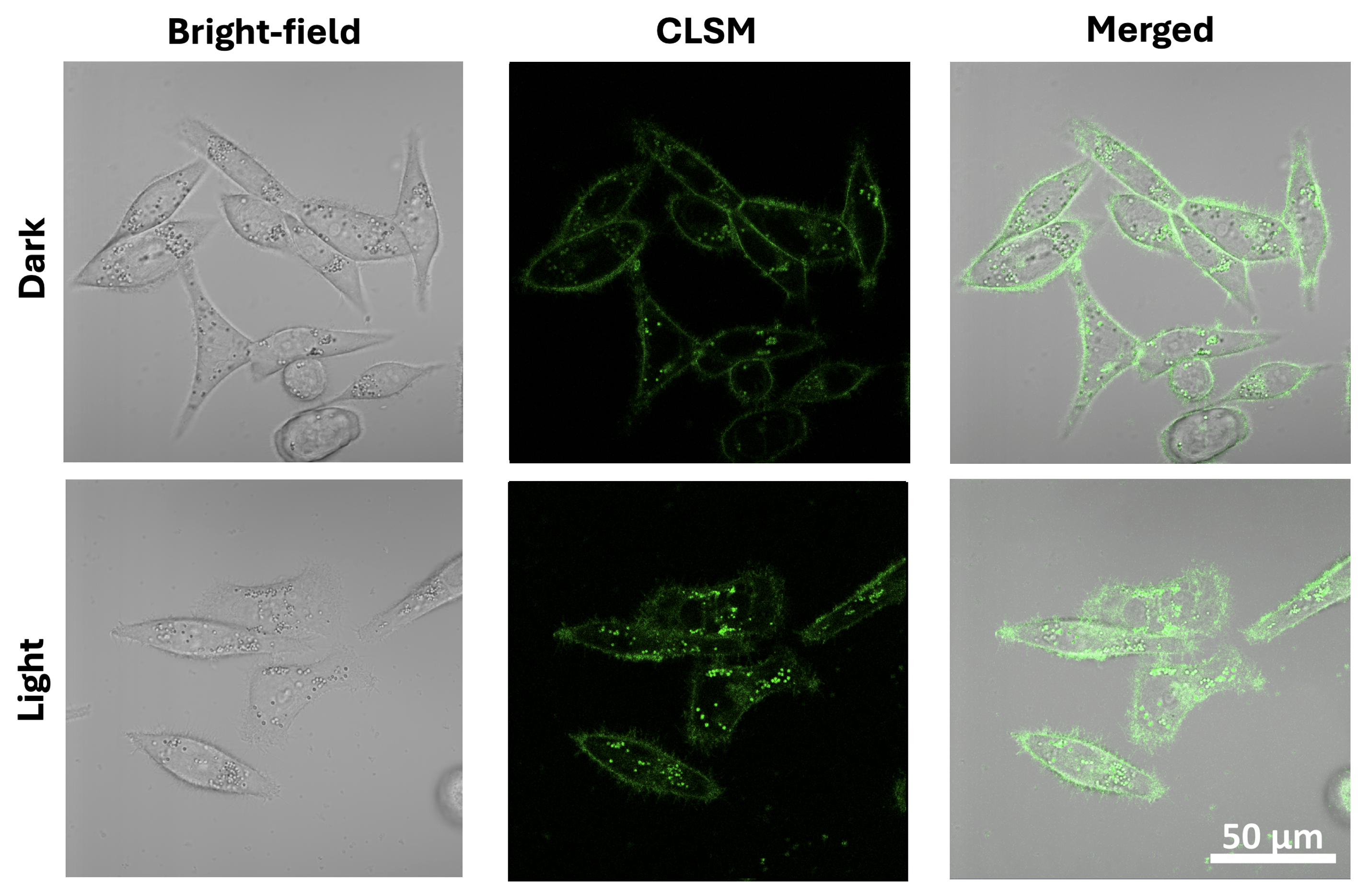


**Figure S24*.*** Bright-field and CLSM images of MCF-7 cells treated with DCFH-DA (0.1 mM) and MF3Ec-TBPP NPs (10 μg·ml^-1^) under white light irradiation (20 mW·cm^-2^) for 30 mins. Prior to staining, MCF-7 cells were incubated under hypoxia for 24 hours to induce hypoxic conditions. λ_ex_ = 488 nm; λ_em_ = 500−550 nm; objective lens = 40×; scale bar = 50 μm.

**Figure S25.** In vitro cytotoxicity of MF3Ec-TBPP NPs in MCF-7 cells. Cell viability was determined via MTT assay incubation with MF3Ec-TBPP NPs (0–20 μg·ml^-1^) under white light irradiation (20 mW·cm^-2^, 40 min). The half-maximal inhibitory concentration (IC_50_) was derived from linear regression analysis. All data shown are expressed as mean values ± standard deviation (Each group, n = 3).


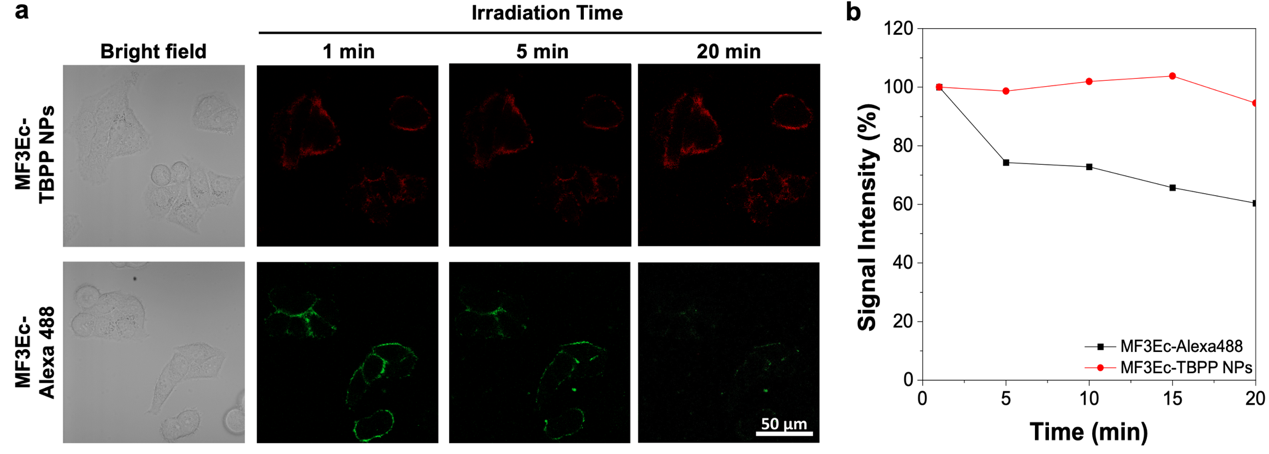


**Figure S26.** Study of photobleaching resistance of TBPP under laser irradiation (a) Bright field and CLSM images of MCF-7 cells stained with MF3Ec-TBPP NPs (10 mg·ml^-1^, *λ*_ex_ = 488 nm, *λ*_em_ = 600-700 nm) or MF3Ec-Alexa 488 (10 mM, *λ*_ex_ = 488 nm, *λ*_em_ = 500–550 nm) under 1, 5 and 20 min of laser irradiation. Objective lens = 40×; scale bar = 50 μm. (b) Plots of fluorescent signal (%) from MCF-7 cells stained with MF3Ec-TBPP NPs or MF3Ec-Alex488 with increasing laser irradiation time.


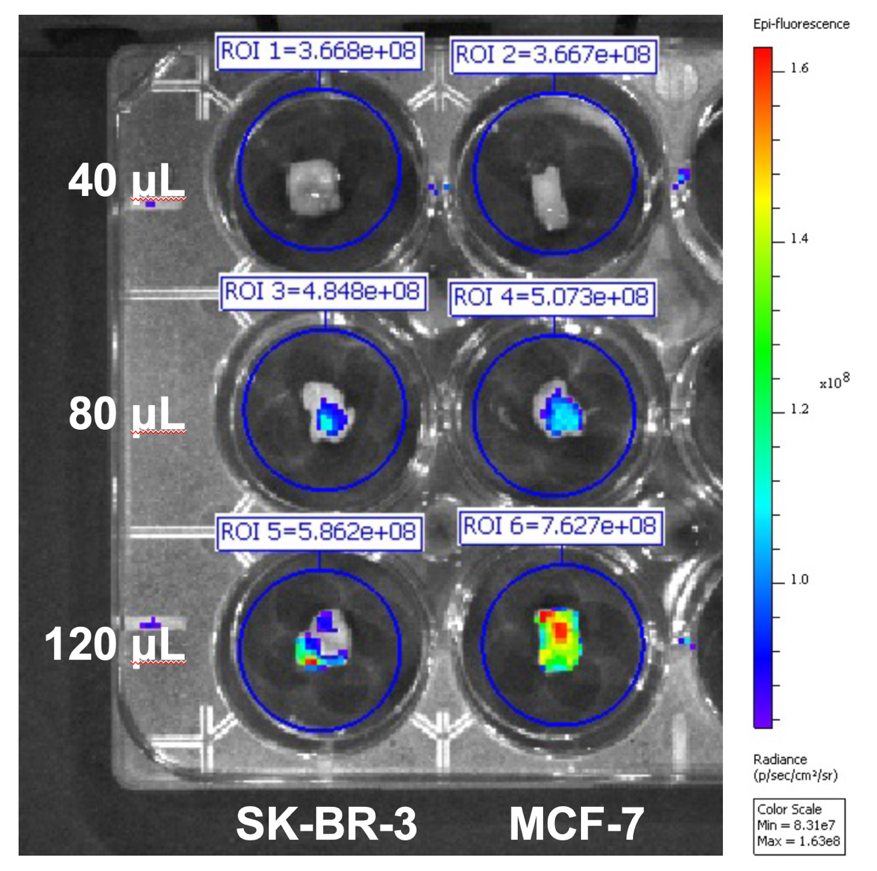


**Figure S27.** *In vitro* fluorescence images of breast cancer tumors. *In vitro* fluorescence images of two subtypes of breast cancer tumors 24 h after injection with different volumes of MF3Ec-TBPP NPs solutions (40, 80 and 120 μL) at the concentration of 0.5 mg·ml^-1^.


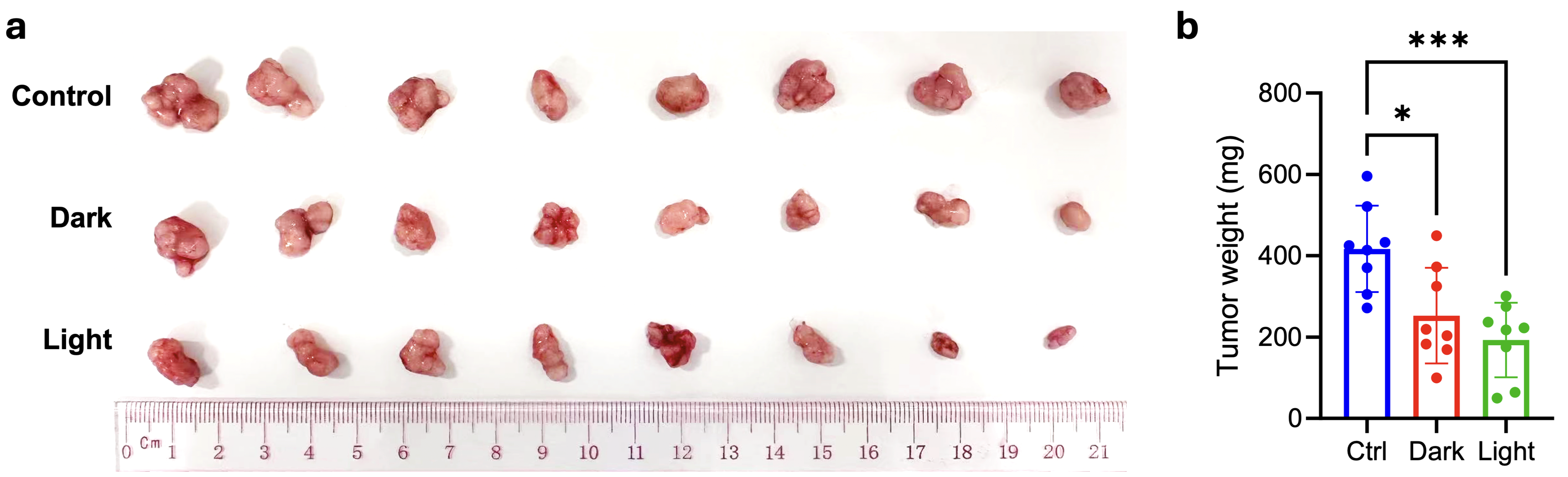


**Figure S28.** (a) Representative images of tumors in mice post-treatment with a 0.5 mg·ml^-1^ MF3Ec-TBPP NPs solution (Each group, n = 12). The terms "dark" and "light" indicate whether the NP-treated groups were not exposed or were exposed to white light (100 mW·cm^-2^ for 30 min), respectively. b) Tumor weight changes in mice recorded at various time points post-NP administration.

~~
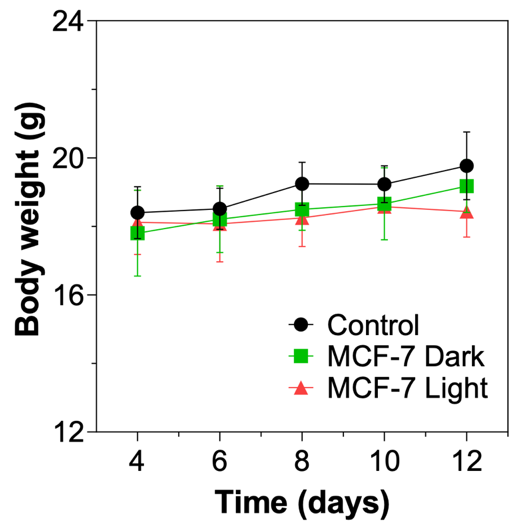
~~

**Figure S29.** Body weight curves of mice at different times within 12 days following MF3Ec-TBPP NPs administration (Each group, n = 3). All data shown are expressed as mean values ± standard deviation.

**
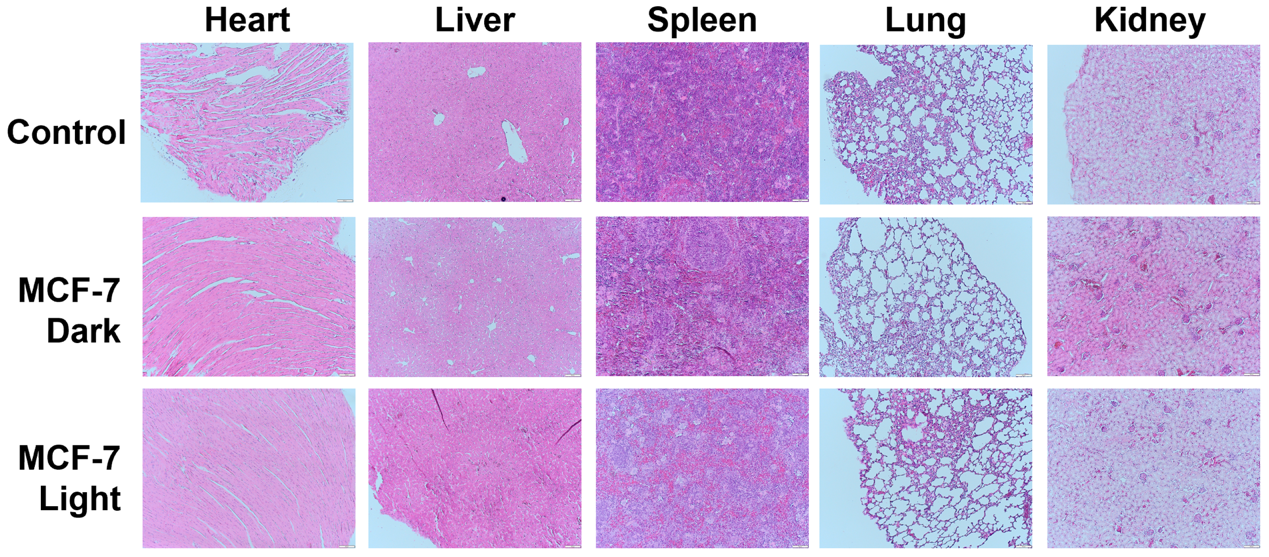
 Figure S30.** H&E staining of major organs’ pathological sections from different groups after 12 -days treatment (Each group, n = 3).


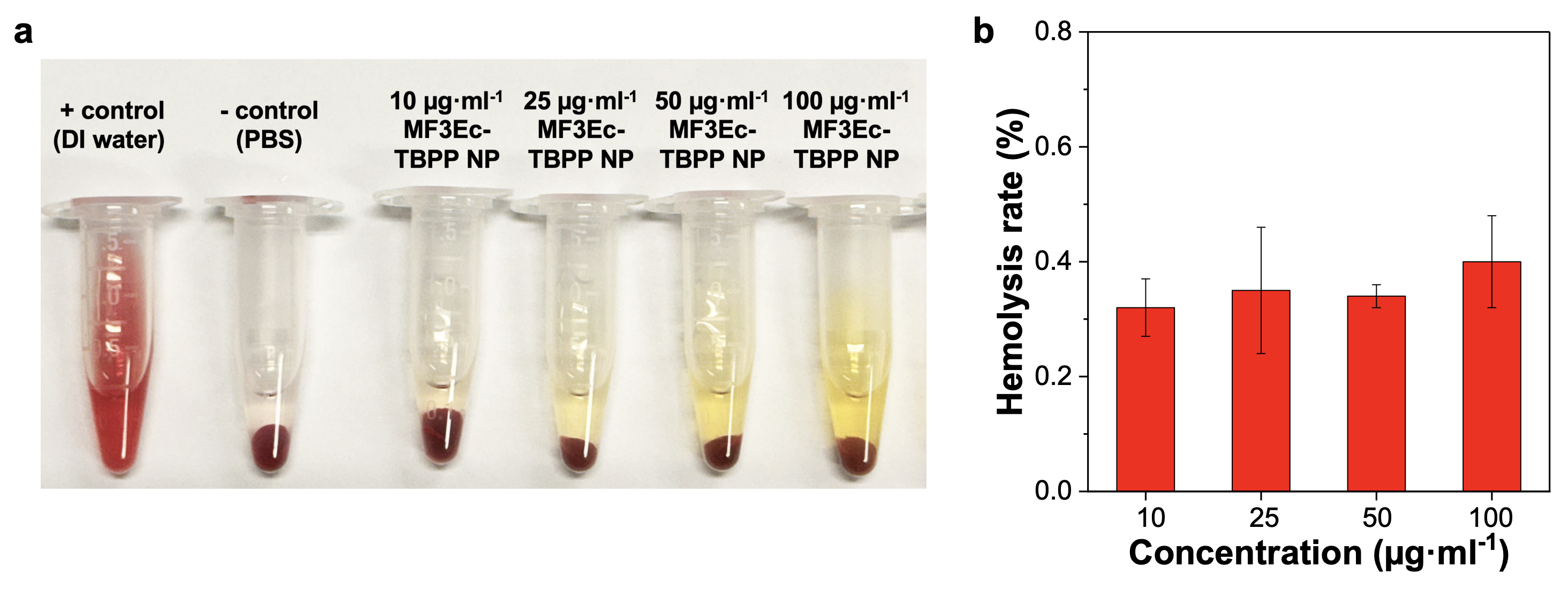
**Figure S31.** Hemolysis assay of MF3Ec-TBPP NPs. (a) Representative image of MF3Ec-TBPP NP solutions at concentrations of 10, 25, 50, and 100 µg·ml^-1^ after centrifugation at 2500 rpm for 5 minutes, showing hemolysis levels under different conditions. Positive control: deionized (DI) water; Negative control: 1× PBS. Samples were incubated with mice blood at 37°C for 1 hour. (b) Quantitative analysis of relative hemolysis rates. Data are presented as mean ± SD (n = 3).


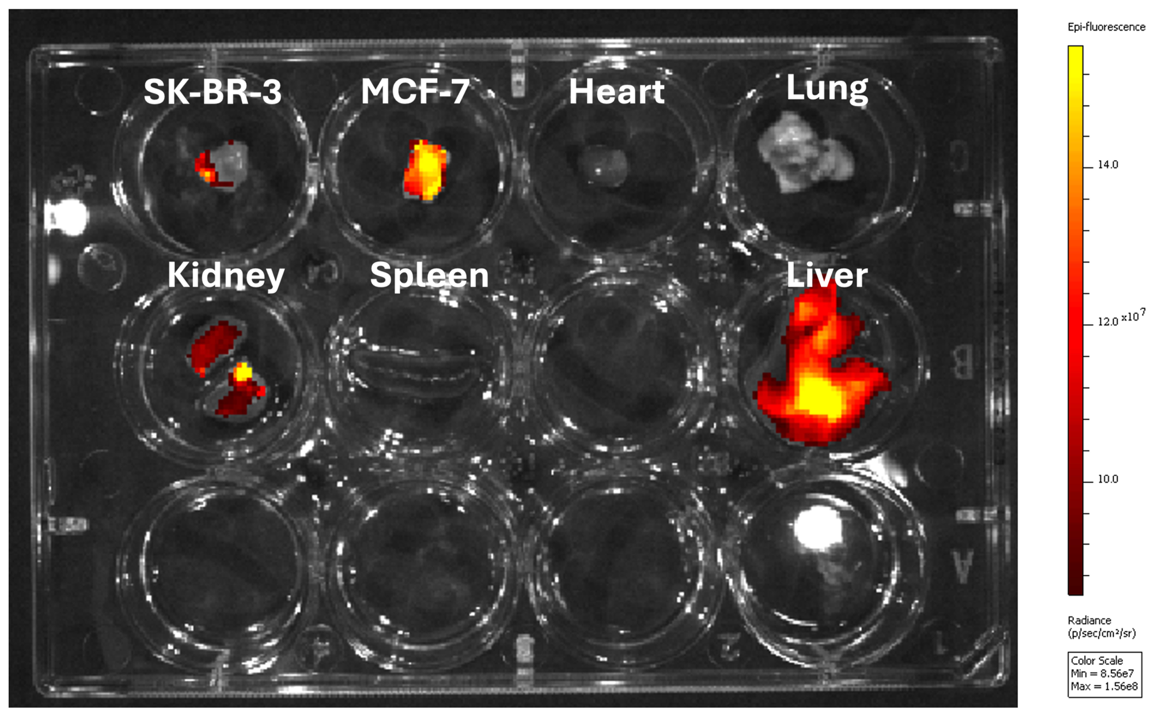


**Figure S32.** Distribution of MF3Ec-TBPP NPs in organs after 48 h. From left to right: SK-BR-3 tumor, MCF-7 tumor, heart, lung, kidney, spleen and liver. λ_ex_ = 500 nm, λ_em_ = 700 nm.

**Table S1**. DNA Sequence of MF3Ec ssDNA aptamers.

| **Aptamer** | **Sequence** |
| --- | --- |
| MF3Ec-NH2 | (5'-NH_2_C12) ACGACCCGATAAGTGCATTAGCACGTC CGAGAAAGGCCAGACGGGTCACACAGAGTTA |
| MF3Ec-Alexa488 | (5'-Alexa488) ACGACCCGATAAGTGCATTAGCACGTC CGAGAAAGGCCAGACGGGTCACACAGAGTTA |

**Supplementary Methods**

**Instruments:** UV-Vis absorption spectra were obtained using a PerkinElmer Lambda 365 Spectrophotometer. Photoluminescence (PL) spectra were captured using an Edinburgh FLS980 spectrofluorometer. Fourier-transform infrared (FTIR) spectra were acquired using a Bruker Vertex 70 FT-IR spectrometer (ATR mode). The morphology of the aggregates was examined using scanning electron microscopy (SEM) with a JEOL JSM-6390, while transmission electron microscopy (TEM) was carried out with a JEOL JEM-2010F for further morphology analysis. NMR spectra (^1^H, ^13^C and ^31^P) were recorded using a Bruker ARX 400 NMR spectrometer. Mass spectrometry was performed on a Water’s Xevo G2-XS Top mass spectrometer in ESI mode. Fluorescent cell imaging was performed using a Zeiss LSM 800 confocal laser scanning microscope.

**Chemical synthesis:** The reagents utilized in the synthesis, including 4-(diphenylamino)phenyl)boronic acid, 4,7-dibromobenzo[c][1,2,5]thiadiazole, tetrakis(triphenylphosphine)palladium, potassium carbonate, toluene, ethanol, dichloromethane, anhydrous magnesium sulfate, hexane, (4-(pyridin-4-yl)phenyl)boronic acid, and tetrahydrofuran (THF), were sourced from suppliers such as Sigma-Aldrich, Meryer, J&K, and Dieckmann. All chemicals were used as received, with the exception of THF and toluene, which were distilled and dried using standard distillation procedures.

**Nanoparticles fabrication:** The materials used for nanoparticle fabrication, including DSPE-PEG-2000, DSPE-PEG-2000-NHS, dipalmitoylphosphatidylcholine (DPPC), cholesterol, DNase-free distilled water, and HEPES buffer, were obtained from suppliers such as Sigma-Aldrich, Meryer, Thermo Fisher Scientific, and Dieckmann. Nanoparticles were fabricated using a QSonica Q125 Sonicator. The MF3Ec-amine and MF3Ec-Alexa 488 conjugates were synthesized and supplied by ATCG Limited, and purified using the HPLC/CE method. All reagents were used without further modification.

**Cell culture:** Cell culture reagents, including Dulbecco’s modified essential medium (DMEM), fetal bovine serum (FBS), and penicillin-streptomycin (PS), were purchased from Thermo Fisher Scientific. Other reagents, such as phosphate-buffered saline (PBS), DCFH-DA, ABDA, HPF, Rose Bengal (RB), and Chlorin e6 (Ce6), were acquired from Sigma-Aldrich. Dihydrorhodamine 123 (DHR), CellMask™ Green Plasma Membrane Stain (CMG-PM), and MTT were also obtained from Thermo Fisher Scientific. Ultrapure water was produced using a Milli-Q Plus System (Millipore Corporation, USA).

**Cell lines**: MCF-7, MDA-MB-231, BT-474 and SK-BR-3 breast cancer cell lines were obtained from the American Type Culture Collection (ATCC, USA).

**Buffers and assays**: 1× Phosphate-buffered saline (PBS, pH 7.4), containing KCl (2.7 mM), NaCl (137 mM), Na₂HPO₄ (10 mM), and KH₂PO₄ (1.8 mM), and HEPES buffer (pH 8.0), consisting of 0.1 M HEPES (4-(2-hydroxyethyl)-1-piperazineethanesulfonic acid) and NaOH for pH adjustment, were purchased from Sigma-Aldrich.

**Synthesis of TBPP:** TBPP was synthesized following a previously reported method. In a round-bottom flask, a mixture of 4-(diphenylamino)phenyl)boronic acid (1.45 g, 5 mmol), 4,7-dibromobenzo[c][1,2,5]thiadiazole (2.93 g, 10 mmol), tetrakis(triphenylphosphine)palladium (578 mg, 0.5 mmol), and K₂CO₃ (2.1 g, 15 mmol) was added to 100 ml of toluene, 20 ml of water, and 10 ml of ethanol. The reaction was refluxed under a nitrogen atmosphere for 12 h. After cooling to room temperature, the reaction mixture was poured into 100 ml of water, followed by extraction with dichloromethane (DCM) and drying over anhydrous magnesium sulfate. The crude product was purified by silica gel column chromatography using a 1:3 (v/v) mixture of DCM and hexane, yielding an orange solid identified as compound 3.

In the next step, compound 3 (500 mg, 1.09 mmol), (4-(pyridin-4-yl)phenyl)boronic acid (261 mg, 1.31 mmol), Pd(PPh₃)₄ (126 mg, 0.109 mmol), and K₂CO₃ (453 mg, 3.27 mmol) were dissolved in 25 ml of tetrahydrofuran (THF) and 5 ml of water. The mixture was heated to 90°C under a nitrogen atmosphere for 18 h. After cooling to room temperature, the solution was extracted with DCM and dried over anhydrous magnesium sulfate. The solvent was evaporated under reduced pressure, and the crude product was purified using silica gel column chromatography with a 10:1 (v/v) mixture of DCM and methanol, affording TBPP as a red solid. ^1^H NMR (400 MHz, Chloroform-d) δ 8.75 (s, 2H), 8.15 (d, J = 8.1 Hz, 2H), 7.98 – 7.79 (m, 6H), 7.67 (d, J = 5.1 Hz, 2H), 7.33 (t, J = 7.7 Hz, 6H), 7.24 (t, J = 8.8 Hz, 4H), 7.11 (t, J = 7.3 Hz, 2H).^13^C NMR (100 MHz, CDCl_3_) δ 149.70, 148.26, 147.41, 131.49, 130.58, 129.96, 129.40, 128.41, 127.29, 127.19, 125.53, 125.01, 123.44, 122.74, 77.34, 77.03, 76.71. MS (MALDI-TOF): m/z 533.1802 [(M)+, calcd 533.1800].

**Fabrication of NHS-TBPP NPs:** A mixture of TBPP (1 mg, 1.88 μmol), DSPE-PEG-2000 (6.8 mg, 2.42 μmol), DSPE-PEG-2000-NHS (1 mg, 0.35 μmol), DPPC (13.8 mg, 18.7 μmol), and cholesterol (3.62 mg, 9.35 μmol) was dissolved in 10 ml of distilled THF. The organic components were then dispersed into 90 ml of DNase-free distilled water using a microtip probe sonicator (12 W) at 50% amplitude with a pulse cycle of 3 s on and 2 s off. During sonication, the THF solution was gradually added dropwise via a 200 µL pipette until a clear solution was formed. The mixture was further sonicated for an additional 5 min. Following sonication, the solution was stirred while compressed air was applied to evaporate the THF, reducing the total volume to 80 ml. The resulting nanoparticles were filtered through a PTFE membrane syringe filter (pore size 0.22 µm). Subsequently, 5 ml of the filtered NHS-TBPP nanoparticles were concentrated using a centrifugal filter unit (Merck Millipore, 50KDa) by centrifugation (Hermle Z 306) at 6000 rpm for 10 min. The nanoparticles were washed with DNase-free distilled water and concentrated to a final volume of 100 µL. The purified NHS-TBPP NPs (0.625 mg·ml^-1^) were stored in the dark at 4°C.

**Synthesis of MF3Ec-TBPP NPs:** 171 µL of DNase-free HEPES buffer (pH = 8.2) was added to resuspend the MF3Ec-NH₂ pellet (17.1 nmol). Then, 78.7 µL of the purified NHS-TBPP NPs solution and 242 µL of DNase-free HEPES buffer (pH = 8.2) were added to the aptamer solution. The reaction mixture was incubated at 4°C for more than 1.5 h. After incubation, the product was purified using a centrifugal filter unit (Merck Millipore, 100 KDa) and washed three times with DNase-free water. The final volume of the purified MF3Ec-TBPP NPs solution was adjusted to 492 µL (0.1 mg·ml^-1^) and stored in a dark environment at 4°C.

**Fabrication of bare TBPP NPs:** A solution of TBPP (1 mg, 1.88 μmol), DSPE-PEG-2000 (6.8 mg, 2.42 μmol), DPPC (13.8 mg, 18.7 μmol), and cholesterol (3.62 mg, 9.35 μmol) was prepared by dissolving the components in 10 ml of distilled THF. The organic phase was then dispersed into 90 ml of DNas-free distilled water using a microtip probe sonicator (12 W) set to 50% amplitude, operating in cycles of 3 s on and 2 s off. The THF solution was added dropwise using a 200 µL pipette during sonication until a clear solution was achieved. The mixture was then sonicated for an additional 5 min. After sonication, the solution was stirred while compressed air was applied to evaporate the THF, reducing the volume to 80 ml. The resulting nanoparticles were passed through a PTFE membrane syringe filter (pore size 0.22 µm). Following this, 5 ml of the filtered NHS-TBPP nanoparticles were concentrated using a centrifugal filter unit (Merck Millipore, 100 KDa) by centrifugation (Hermle Z 306) at 6000 rpm for 6 min. The nanoparticles were washed with DNase-free distilled water and concentrated to a final volume of 100 µL. The purified NHS-TBPP nanoparticles (0.625 mg·ml^-1^) were stored in the dark at 4°C.
